# Supplementary material for: The osteology and affinities of Eotyrannus lengi, a tyrannosauroid theropod from the Wealden Supergroup of southern England
Source: PeerJ. 2022 Jul 7;10:e12727. doi: 10.7717/peerj.12727 (PMC9271276; doi:10.7717/peerj.12727)
Supplement: Supplemental Information 2 — List of the 1145 phylogenetically informative characters included in the data set. Character statement numeration follows the complete list, from Lee et al. (2014) and Cau et al. (2015). [file peerj-10-12727-s002.doc]

**Character statements**

1): Skull, anteroposterior length in adult: less than (0); more than (1) 4/5 of femoral length. The length of the skull is here defined as the distance from the anteroventral margin of premaxilla to the posteroventral margin of the mandibular condyles of the quadrate.

3): Premaxilla, body in front of the external naris, angle between the anterior margin and the alveolar margin: more than 75º (0); less than 75º (1). (Modified from Rauhut 2003).

5): Premaxilla, shape of internarial process: transversely flattened (0); dorsoventrally flattened (1). (Sereno 1999).

8): Premaxilla, subnarial process, posteriormost extent relative to posteroventral corner of external naris: posterior or coincident (0); anterior (1). (Modified from Rauhut 2003).

10): Premaxilla, anterior half of oral margin, teeth: present (0); absent (1).

11): Premaxillae in adult: unfused (0); fused (1). (Sereno 1999).

12): Premaxilla, subnarial body, proportions in lateral view: not taller than long (0); taller than long (1). (Modified from Holtz 2000).

13): Premaxilla-maxilla articulation, lateral surface, exposed subnarial foramen: absent (0); present (1). (Gauthier 1986; Langer and Benton 2006).

14): Premaxilla, fifth alveolus: absent (0); present (1).

15): Premaxillary teeth, serration: present at least in posterior teeth (0); absent in all teeth (1). (Modified from Sereno 1999).

16): Premaxillary teeth, crown cross-section shape: round-elliptical (0), asymmetrical (more convex labially, more flattened lingually) (1). (Modified from Holtz et al. 2004).

17): Premaxilla, buccal margin in lateral view: in the same plane as maxilla (0), set below the anterior buccal margin of the maxilla (1).

18): Premaxilla, medial palatal alae, development: widely contacting in front of vomers (0), reduced and separated (1). (Modified from Carrano and Sampson 2008; Holtz 2000).

20): Premaxillary teeth, first tooth size: comparable to (0); larger than (1) the remaining premaxillary teeth. (Modified from Senter 2010).

21): Maxilla, promaxillary recess/fenestra: absent (0); present (1). (Holtz 2000).

22): Maxilla, maxillary recess/fenestra: absent (0); present (1). (Gauthier 1986).

24): Maxilla, ventral process, lateral horizontal ridge bounding the antorbital fossa: absent (0), present (1). (Carrano and Sampson 2008).

25): Nasal, narial fossa: absent (0); present (1). (Rauhut 2003; Wilson et al. 2003; Eddy 2008).

26): Nasal, narial margin, distance from narial border of premaxilla: no more than 1/3 (0); more than 1/3 (1) of preorbital skull length. (Modified from Holtz et al., 2004).

27): Premaxilla, narial margin, anteroventral corner, position relative to the mid-point of premaxillary oral border: anterior (0); posterior (1). (Modified from Rauhut 2003).

28): Maxilla, participation to the ventral margin of the external naris: absent (0); present (1).

29): Maxilla, palatal process, mediolateral development: narrow ridges (0); broad shelves (1).

31): Maxilla, antorbital fossa, anterior margin, anteroposterior extent: more than 1/5 (0); less than 1/5 (1) of antorbital fossa length. (Modified from Holtz 2000; Carrano and Sampson 2008; Wilson et al. 2003).

32): Maxilla, antorbital fossa, ventral margin, anteroposterior extent: more than (0); less than (1) 1/3 of the length of the antorbital fenestra. (Modified from Yates 2006).

33): Maxillary teeth ventral to orbit and ascending ramus of lacrimal: present (0); absent (1). (Modified from Gauthier 1986; Holtz 2000).

34): Cheek teeth (estimated sum of dentary and maxillary teeth), number: no more than 75, relatively large alveoli (0); more than 75, relatively small alveoli (1).

35): Maxilla, preantorbital process (anterior to antorbital fossa), length: subequal or less (0); more than (1) 2/5 of the length of the maxilla. (Modified from Sereno 1999).

36): Maxilla, anterodorsal margin, shape in lateral view: straight to convex (0); concave (1). (Modified from Rauhut 2003; Holtz et al. 2004).

37): Maxilla, dorsal process, posterodorsal extent dorsal to antorbital fossa: elongate and posteriorly directed (0); strongly reduced (1). (Modified from Carrano and Sampson 2008).

38): Maxilla, ventral process, jugal overlap: less than (0); more than (1) 1/3 of maxillary length. (Modified from Wilson et al. 2003).

39): Maxilla, antorbital fossa, anteroventral margin: with (0); without (1) a raised rim. (Senter 2010).

41): Maxilla, lateral lamina obscuring the anteriormost portion of the antorbital fossa in lateral view: absent (0), present (1). (Holtz et al. 2004).

42): Nasal, narial margin, position: anterior (0), posterior (1) to the anterior margin of the maxillary antorbital fossa.

43): Nasal, participation to the antorbital fossa: absent (0); present (1). (Rauhut 2003).

44): Premaxilla/maxilla/dentary, lateral surface, extensive pattern of grooves: absent (0); present (1). (Modified from Wilson et al. 2003).

45): Nasal, median crest/eminence: absent (0); present (1). (Modified from Holtz 2000; Rauhut 2003).

46): Nasal, dorsal surface: smooth (0); rugose (1). (Holtz et al. 2004).

47): Nasals: apneumatic (0); pneumatized (1). (Rauhut 2003).

48): Nasals, dorsal view: expanded posteriorly, so that the lateral margins diverge (0), width subequal throughout their length (1). (Holtz 2000).

50): Nasal, subnarial process: present (0); absent (1). (Sereno 1999).

51): Nasal, dorsal surface, row of foramina: absent (0); present (1). (Carrano and Sampson 2008).

53): Prefrontal, participation to the anterior margin of orbit: present (0); absent (1). (Modified from Rauhut 2003).

54): Frontal-lacrimal contact: absent (0); present (1). (Modified from Gauthier 1986; Rauhut 2003).

55): Lacrimal, orbital margin, suborbital process: absent (0); present (1). (Currie and Carpenter 2000).

56): Lacrimal, ventral process, main axis in lateral view: anterodorsal (0); subvertical or slightly posterodorsal (1).

57): Lacrimal, posterodorsal corner, pneumatic recesses: absent (0); present (1). (Holtz 2000).

58): Jugal, anterior process, shape: unexpanded anteriorly (0); dorsoventrally expanded anteriorly (1). (Rauhut 2003).

59): Lacrimal, laterodorsal shelf bordering the antorbital fossa: absent (0); present (1). (Holtz 2000).

60): Lacrimal, anterodorsal process, length: more than 2/5 (0); less than 2/5 (1) of the lacrimal height. (Modified from Holtz 2000; Carrano and Sampson 2008).

61): Lacrimal, posterodorsal process: absent (0); present (1). (Senter 2010).

62): Lacrimal, anteroposterior length of the ventral process (at base) in lateral view: less than 1/3 (0); more than 1/3 (1) of the lacrimal height.

63): Jugal, participation to the antorbital fossa: absent (0); present (1). (Holtz 2000).

64): Lacrimal, ventral process, position of anterior half of the margin: above the ventral margin of the orbit (0); at the level of ventral margin of the orbit (1). (Modified from Rauhut 2003).

65): Jugal, postorbital process, height: more (0); less (1) than half orbit height. (Modified from O’Connor 2009).

67): Jugal, anterior pneumatic recess: absent (0); present (1). (Holtz 2000; Rauhut 2003).

68): Lacrimal, laterodorsal recess, position: exposed laterally (0); in an anteriorly facing recess (1).

69): Infratemporal fenestra, ventral margin length: more than (0); less than (1) half orbit height.

71): Squamosal, distal margin of the quadratojugal process: closer to the posterior margin of the infratemporal fenestra (0); closer to the anterior margin of the infratemporal fenestra (1).

72): Frontal, shape of the lateral margin in dorsal view: describes a smooth transition between the anterior half and the postorbital process (0); describes an abrupt transition between the anterior half and the postorbital process (1). (Senter 2010).

73): Frontal, dorsal surface, supratemporal fossa: absent (0); present (1). (Modified from Sereno 1999; Norell et al. 2001).

75): Frontal, degree of thickening: reduced, bone laminar in cross section (0); marked, bone dorsoventrally expanded (1). (Wilson et al. 2003).

76): Frontal, shape of the anterior half in dorsal view: trapezoidal (0); triangular (1). (Modified from Holtz 2000; Wilson et al. 2003).

77): Frontal, ventral surface, medial delimitation of the orbit: not expanded ventrally (0); expanded ventrally, forming a pronounced rim (1). (Modified from Rauhut 2003).

78): Parietal, length: less than 3/4 of the frontal (0), subequal or more than 3/4 of the frontal (1).

79): Parietal, dorsal surface, sagittal crest: absent (0), present (1). (Rauhut, 2003).

80): Parietals: unfused (0); fused (1).

81): Fronto-parietal fusion: absent (0); present (1). (Coria and Currie 2002).

82): Frontals, mediolateral width of the paired bones: less than (0); more than (1) 4/3 of frontal length.

83): Parietal, posterodorsal projection (nuchal plate): absent (0); present (1). (Wilson et al. 2003).

84): Squamosal, participation to the supratemporal fossa: absent (0); present (1). (Hwang et al. 2004; Benson et al., 2010).

85): Squamosal, participation to the dorsal margin of the infratemporal fenestra: subequal to the postorbital participation (0); wide, postorbital process of the squamosal anteriorly expanded, reduced postorbital participation to the dorsal margin of the infratemporal fenestra (1).

86): Squamosal, lateroventral (quadratojugal) process, ratio of proximodistal length to mid-length anteroposterior diameter: less than (0); more than (1) 3 times.

87): Squamosal, posterolateral shelf: absent (0); present and overhanging the quadrate head (1). (Senter 2010).

88): Postorbital, anterodorsal process, contact with the preorbital bar: absent (0); present (1). (Brusatte and Sereno 2008).

89): Postorbital, posterodorsal process: subequal or less than the anterodorsal process of the postorbital (0); longer than the anterodorsal process of the postorbital (1).

90): Postorbital, frontal (anterodorsal) process: dorsoventrally unexpanded, lacking external skulpturing (0); dorsoventally expanded and rugose (1). (Modified from Wilson et al. 2003; Currie and Carpenter 2000).

91): Postorbital, suborbital process bordering ventrally the eyeball: absent (0); present (1). (Modified from Wilson et al. 2003; Currie and Carpenter 2000).

92): Postorbital, ventral (jugal) process, cross section: subtriangular, longer than deep (0); “U”-shaped and transversely expanded (1). (Sereno 1999).

93): Postorbital, ventral (jugal) process, contribution to the posterior margin of the orbit: more than 1/2 (0); less than 1/2 (1) of the orbital margin.

94): Quadrato-articular articulation, anteriormost extent relative to the dorsoventral plane of the occipital condyle: ventral to posterior (0); anterior (1). (Modified from Holtz 2000; Rauhut 2003).

95): Quadrato-articular articulation, ventralmost extent: lies at the same level of the dorsal margin of the dentary (0), lies well below the dorsal margin of the dentary, at or close to the level of the ventral margin of the dentary (1). (Modified from Gauthier 1986).

96): Quadrate, posterior margin in lateral view: exposed (0), hidden by quadratojugal (1).

97): Quadrate shaft, posterior surface, pneumatic recess: absent (0); present (1). (Senter 2010).

98): Quadratojugal, participation to the quadrate foramen: extensive, comparable to the quadrate (0); reduced (foramen enclosed almost entirely within the dorsal process of the quadrate) (1). (Modified from Rauhut 2003).

100): Quadrate, quadrate foramen margin, placement: a mid-height or dorsal (0); ventral, close to mandibular condyles (1). (Loewen et al. 2013).

101): Quadrate, distal end, lateral condyle, width: subequal in size or larger (0); narrower (1) than the mediodistal condyle. (Coria and Salgado 2000).

102): Quadrate, pterygoid process, dorsoventral expansion: reduced, reaches its greatest anteroposterior width dorsally to the mid-point of the dorsoventral axis of the quadrate (0); expanded, reaches its greatest anteroposterior width at the same level or ventrally to the mid-point of the dorsoventral axis of the quadrate (1).

104): Quadratojugal, articulation with the squamosal: present (0); absent (1). (Modified from O’Connor 2009).

105): Quadratojugal, anteroventral (jugal) process, anteriormost extent in lateral view: does not (0); does (1) reach the level of the postorbital bar. (Modified from Holtz et al. 2004).

106): Quadratojugal, dorsal (squamosal) process in lateral view: slender (anteroposterior length at mid-height less than 1/3 of the length of the jugal process of the quadratojugal) (0); broad (anteroposterior length at mid-height longer than 1/3 of the length of the jugal process of the quadratojugal) (1). (Modified from Holtz et al. 2004).

108): Quadratojugal, posteroventral process: absent (0); present (1). (Modified from Holtz et al. 2004).

110): Supraoccipital, dorsoventral diameter in occipital view: less than twice the dorsoventral diameter of the foramen magnum (0); more than twice the dorsoventral diameter of the foramen magnum (1). (Wilson et al. 2003).

111): Parietal, tongue-like process overlapping the supraoccipital: absent (0); present (1). (Coria and Currie 2002).

112): Foramen magnum, shape: subcircular (0); elliptical, taller than wide (1).

114): Occipital condyle, ventrolateral surface, pair of pneumatic cavities that join medially: absent (0); present (1). (Coria and Currie 2002).

115): Occipital condyle, angle formed with the basituberal process in lateral view: perpendicular or almost perpendicular (0); acute (1). (Coria and Currie 2002).

116): Basioccipital, participation to the basal tubera: present (0); absent (1). (Holtz 2000).

117): Basal tubera, mediolateral width between the paired processes: more than the mediolateral width of the occipital condyle (0); less than the mediolateral width of the occipital condyle (1). (Modified from Holtz 2000; Senter 2010).

118): Paroccipital processes, ventral rim of the base, position: above or level with the dorsal border of the occipital condyles (1); situated at or below mid-height of the occipital condyles (1). (Rauhut 2003).

119): Parabasisphenoid, anterior tympanic recess: absent (0); present (1). (Rauhut 2003).

120): Posterior tympanic recess: absent (0); present (1).

121): Paroccipital processes, distal end, placement relative to foramen magnum: at the same level or dorsally (0); ventrally (1). (Modified from Holtz 2000; Currie and Carpenter 2000).

122): Forebrain: cylindrical and unexpanded (0); expanded and trapezoidal in dorsal view, leaves marked impressions on the inner surface of the neuroanterior bones (1). (Modified from Rauhut 2003).

123): Bulbous parasphenoid capsulae: absent (0); present (1). (Holtz 2000).

124): Pneumatic recess ventral to fenestra ovalis (subotic recess): absent (0); present (1).

126): Basisphenoid, ventral recess: absent (0); present (1). (Holtz 2001; Rauhut 2003).

127): Basisphenoid: antero-posteriorly elongate (0); dorso-ventrally expanded (1). (Modified from Sereno 1999).

128): Basal tubera, notch separating from the exoccipital-opisthotic and the basisphenoid: absent (0); present (1). (Holtz et al. 2004).

129): Basipterygoid process, shape: anteroposteriorly short and fingerlike (approximately as long as wide) (0); elongate anteroposteriorly (longer than wide) (1). (Modified from Holtz et al. 2004; Senter 2010).

130): Basipterygoid processes, medio-lateral width: more than the mediolateral width across the basal tubera (0); less than the mediolateral width across the basal tubera (1). (Holtz 2000).

131): Basipterygoid process: solid (0); hollow (1). (Senter 2010).

132): Otosphenoid crest: vertical on the basisphenoid and prootic and does not border an enlarged pneumatic recess (0), well developed, crescentic, and thin, forming the anterior edge of the enlarged pneumatic recess (1). (Senter 2010).

133): Prootic, depression for pneumatic recess: absent (0); present as dorsally open (1). (Modified from Senter 2010).

134): Cranial nerve V, number: single (0); two (1). (Modified from Currie and Carpenter 2000).

136): Exit of the cranial nerves X and XI, position on braincase: laterally (0); posteriorly through a foramen lateral to the exit of cranial nerve XII and the occipital condyle (1). (Modified from Rauhut 2003; Senter 2010).

139): Pterygoid in ventral view; shape of the lateroposterior border, at the confluence of the quadrate process and the mandibular process: narrow incisure facing posteriorly (0); wide concavity facing laterally (1).

140): Palatines, contact medially: absent (0); present (1). (Currie and Carpenter 2000).

141): Palatine, pneumatic recess at the confluence of the maxillary process and of the vomeropterygoideus process: absent (0); present (1). (Currie and Carpenter 2000).

142): Pterygoid, accessory fenestra with the palatine: absent (0); present (1). (Gauthier 1986; Rauhut 2003).

145): Ectopterygoid, ventral recess: absent (0); present (1). (Modified from Rauhut 2003).

146): Ectopterygoid and palatine, contact: absent (0); present (1). (Modified from Holtz 2000).

150): Dentary, symphysis, shape in lateral view: subtriangular (0); quadrangular, with a ventrodistally expanded tip (1). (Modified from Currie and Carpenter 2000; Brusatte and Sereno 2008).

151): Dentary, second alveolus, size compared to alveoli fourth to sixth: comparable (0); much smaller (1).

152): Dentary, ventral margin, shape in lateral view: straight to convex (0); concave (1). (Modified from Senter 2010; Sereno 1999).

153): Paradental laminae, plates: distinct (0); indistinct (1). (Modified from Senter 2010).

154): Maxillary and dentary teeth, distal margin, apicobasal curvature in labial/lingual view: marked (the apex of the tooth is placed distally to the distal margin of the crown base, the distal margin is concave) (0); reduced (the apex of the tooth is placed above the crown base, the distal margin is straight or convex) (1). (Modified from Sereno 1999).

155): Dentary, anterior fourth: toothed (0); lacking teeth (1).

156): Dentary, post-symphyseal region (excluded the part included in the character 155): toothed (0); lacking teeth (1).

157): Dentary, interdental septa, mesiodistal diameter relative to adjacent alveoli: smaller to subequal (0); much larger, teeth widely spaced (1). (Modified from Sereno 1999; Holtz 2000; Senter 2010).

158): Maxillary teeth, apicobasal length of the longest crown: less than (0); more than (1) 6/5 to the depth of the ventral ramus of maxilla. (Modified from Rauhut 2004).

159): Maxillary/dentary teeth, carinae, serration: present at least along distal carina (0); absent, carinae unserrated (1). Inapplicable in taxa lacking carinae. (Modified from Senter 2010, O’Connor 2009).

160): Maxillary and dentary teeth, basal constriction between the root and the crown: absent (0); present (1). (Holtz 2000).

161): Maxillary/dentary teeth, mesial/distal carinae: present (0); absent (1). (Modified from Senter 2010; O’Connor 2009).

163): Teeth, mesial/distal denticulation, development: small denticles (0); coarse serration (1). (Modified from Senter 2010).

164): Dentary, third alveolus, size compared to alveoli fourth to sixth: subequal (0); clearly larger (1). (Modified from Gauthier 1986).

165): Teeth, surfaces adjacent to carinae: smooth (0), having series of marked wrinkles, inclined basally (1). (Currie and Carpenter 2000).

166): Dentary, posteroventral process, participation to the ventral border of the external mandibular fenestra: less than (0); more than (1) the angular participation.

167): Dentary, posterodorsal process: absent (0); present (1). (Modified from Holtz 2000).

169): Dentary, anterior symphysis, shape in dorsal/ventral view: “V”-shaped (0); “U”-shaped (1). (Senter 2010).

170): Dentary, dorsolateral surface, longitudinal posterior ridge/shelf: absent (0); present (1). (Modified from Senter 2010; Barrett 2009).

171): External mandibular fenestra, anteroposterior diameter: less than 1/4 of the length of the mandible (0); subequal or longer than 1/4 of the length of the mandible (1).

172): External mandibular fenestra, dorsoventral diameter: more (0); less (1) than 1/3 of the maximus dorsoventral diameter of the mandible.

174): Retroarticular process, width in dorsal view: less (0); more (1) than the width of the pre-glenoideal region of the mandible. (Modified from Rauhut 2003).

175): Retroarticular process, attachment of the M. depressor mandibulae, inclination: facing posterodorsally (0); facing almost completely posteriorly (1). (Sereno 1999; Rauhut 2003).

176): Retroarticular process, anteroposterior length: longer than the dorsoventral depth of the mandible at the level of the mandibular glenoid (0); less than the dorsoventral depth of the mandible at the level of the mandibular glenoid (1). (Modified from Senter 2010).

177): Dentary-surangular articulation in lateral view: surangular overlaps dentary (0); dentary overlaps surangular (1).

178): Dentary, lateral surface, posterior groove: shallow (0); deep with distinct margins (1). (Modified from Senter 2010).

179): Splenial, lateroventral exposition in lateral view: absent, covered by deep posteroventral process of dentary (0); present, posteroventral process of dentary shallow (1). (Senter 2010).

180): Splenial, posterior margin in lateral/medial view: straight or slightly concave posteriorly (0); deeply concave posteriorly (1). (Rauhut 2003).

181): Coronoid ossification: large to moderately developed (0); reduced as a split of bone (1). (Modified from Senter 2010).

182): Articular, pneumatic recess: absent (0); present (1). (Modified from O’Connor 2009).

184): Splenial, mylohyoid anteroventral foramen/notch: absent (0); present (1). (Rauhut 2003; Sereno 1999).

185): Surangular, position of the anteriormost extent: anterior to or at the level of the external mandibular fenestra (0); posterodorsal to the anterior margin of the external mandibular fenestra (1). (Carrano and Sampson 2008).

187): Surangular, coronoid process: absent (0); present (1). (Modified from Norell et al. 2001). 188): Surangular, posterolateral foramen/fenestra: small, maximum diameter less than (0); expanded, maximum diameter more than (1) 1/3 of the anteroposterior length of the mandibular glenoid (1). (Modified from Holtz et al. 2004).

189): Surangular, maximus dorsoventral depth of the anterior process: less than twice maximus depth of the angular (0); more than twice maximus depth of the angular (1). (Holtz 2000).

191): Surangular, participation to the mandibular glenoid: present (0); absent (1). (Modified from Maryańska et al. 2002).

193): Angular, anterior prong, contact with the dentary-splenial cavity: absent (0); present (1). (Holtz 2000).

194): Presacral vertebrae, cervical and anterior dorsal centra, anterior surface, shape: flat to concave (0); convex (1). (Modified from Rauhut 2003; Holtz 2000; Carrano and Sampson 2008).

195): Cervical ribs, pneumatic recess: absent (0); present (1). (Wilson et al. 2003).

196): Cervical ribs, articulation to vertebrae in adults: loose (0); firmly attached/fused (1). (Holtz 2000).

197): Atlas, neurapophyses, shape in lateral view: subrectangular and posterodorsally directed (0); subtriangular (1). (Currie and Carpenter 2000).

198): Axis, neural spine in lateral view: low and antero-posteriorly expanded (0); mediolaterally compressed and dorso-ventrally elongate (1). (Currie and Carpenter 2000).

199): Axis, neural spine base, large groove/excavation: present (0); absent (1). (Rauhut 2003).

200): Axis, diapophyses, development: poorly developed (0); prominent (1). (Modified from Tykoski 2005; Holtz 2000).

201): Axis, pleurocoel: absent (0); present (1). (Rauhut 2003; modified from Tykoski 2005).

202): Axis, ventral keel: present (0); absent (1). (Currie and Carpenter 2000).

204): Axis, intercentrum, position of the ventral margin: at the same level of the ventral margin of the axial centrum (0); strongly tilted dorsally (1). (Currie and Carpenter 2000).

205): Axis, neural spine, dorsal surface, shape: mediolaterally narrow (0); mediolaterally expanded (1). (Currie and Carpenter 2000; Holtz 2000; Brusatte et al. 2010).

206): Cervical vertebrae, anterior post-axial centra, shape in anterior view: as wide as tall (0); dorsoventrally compressed (1). (Modified from Holtz 2000).

207): Cervical vertebrae, anterior post-axial centra, ventral keel, development: absent (0); present (1). (Ezcurra and Novas 2007).

208): Postaxial cervical vertebrae epipophyses: absent (0); present (1). (Rauhut 2003).

209): Postaxial cervical prezygoepipophyseal lamina: absent (0); present (1). (Modified from Carrano et al. 2002).

210): Cervical vertebrae, post-axial centra, ventral sulcus delimited by ventrolaterally directed ridges: absent (0); present (1). (Makovicky et al. 2005).

211): Cervical vertebrae, anterior post-axial neural arches, neural spines, length: longer than 1/2 of the length of the neural arch (0); subequal or less than 1/2 of the length of the neural arch (1).

212): Cervical vertebrae, anterior post-axial neural spines in lateral view: longer than tall (0); taller than long (1).

213): Cervical vertebrae, post-axial neural arches, position of the neural spine: placed at mid-length or in its posterior half (0); placed in the anterior half (1). (Carrano and Sampson 2008).

214): Cervical vertebrae, post-axial neural spines, shape in anterior/posterior view: dorsoventrally taller than mediolaterally wide (0); wider than tall (1). (Coria and Salgado 2000).

215): Presacral vertebrae, neural arches, postzygodiapophyseal lamina, development: poorly developed (0); pronounced (1). (Coria and Salgado 2000).

216): Cervical vertebrae, anterior post-axial prezygapophyses, mediolateral placement in anterior view: dorsally to the centrum (0); laterally to the lateral border of the centrum (1). (Modified from Senter 2010).

217): Cervical vertebrae, articular surface, anterior post-axial prezygapophyses, shape: straight (0); anteroposteriorly convex, distal half flexed ventrally (1). (Holtz 2000; Rauhut 2003).

218): Presacral vertebrae, neural arches, prespinal fossa, development: shallow depression (0); dorsoventrally elongate and deep (1). (Modified from Wilson et al. 2003).

219): Cervical vertebrae, posterior centra, anterior surface, elevation relative to the posterior surface: reduced (0); marked (1). (Gauthier 1986).

220): Presacral vertebrae, anterior ribs, alariform process: absent (0); present (1). (Wilson et al. 2003).

221): Cervical ribs, length: longer than their corresponding centra (0); subequal or less than their corresponding centra (1).

222): Cervical vertebrae, middle and posterior centra, length: less than two times (0); more than two times (1) the dorsoventral height of the anterior surface. (Modified from Holtz 2000; Wilson et al. 2003).

223): Cervical vertebrae, post-axial centra, position of the longest centrum: proximal to the VI position (0); distal to the VI position (1). (Gauthier 1986).

224): Presacral vertebrae, neural arches, pneumatic recesses on the lateroventral surface, development: poorly developed (0); pronounced (1). (Modified from Carrano and Sampson 2008; Wilson et al. 2003).

225): Dorsal vertebrae, anterior centra, ventral processes anterior to the keel (hypapophysis): poorly developed (0); strongly developed (1). (Modified from Rauhut 2003; O’Connor 2009).

226): Dorsal vertebrae, anterior centra, ventral keel, development: poorly developed (0); pronounced (1). (Rauhut; 2003).

227): Anterior presacral centra, posterior half of centrum, pneumatic recess: absent (0); present (1). (Modified from Holtz 2000).

228): Dorsal vertebrae, anterior centra, pleurocoels: absent (0); present (1). (Holtz 2000; Currie and Carpenter 2000; Senter 2010).

229): Presacral vertebrae, pleurocoel, structure: camerate (few diverticula separated by robust septa) (0); camellate (many diverticula separated by thin lamellae) (1). (Modified from Holtz 2000; O’Connor 2006).

230): Dorsal vertebrae, centra, anterior surface, proportions: as tall as wide (0); mediolaterally wider than dorso-ventrally tall (1).

232): Dorsal vertebrae, posteriormost centra, anteroposterior length: subequal or more (0); less (1) than anterior centrum height. (Modified from Rauhut 2003).

235): Dorsal vertebrae, transverse processes, anterior margin, orientation: laterally directed (0); strongly backturned posterolaterally (1). (Modified from Tykoski 2005; Holtz 2000).

236): Dorsal vertebrae, posterior neural spines, dorsal margin, anteroposterior expansion relative to spine length at mid-height: moderate (0); marked (1). (Modified from Senter 2010).

237): Dorsal vertebrae, posterior neural spines, dorsoventral axis, inclination in lateral view: perpendicular to the anteroposterior axis of the centrum or posterodorsally inclined (0); anterodorsally directed (1). (Currie and Carpenter 2000).

238): Dorsal vertebrae, parapophyses, shape: low processes (0); elongate, often stalked on pedicels (1). (Carrano and Sampson 2008; Senter 2010).

240): Dorsal vertebrae, posteriormost parapophyses, position: anteroventral or anterior to transverse processes (tuberculum and capitulum of the ribs offset horizontally) (0); distinctly ventral to transverse process (tuberculum and capitulum of the ribs offset vertically) (1). (Rauhut 2003).

242): Dorsal ribs, ventral process: unossified (0); ossified (1).

244): Dorsal vertebrae, zygapophyses, position: abutting one another dorsal to the neural canal (0); placed laterodorsal to neural canal (1). (Senter 2010).

247): Dorsal vertebrae, hyposphene, development: hyposphene developed as a single sheet of bone (0); hyposphene wide, formed by the ventrally bowed medial parts of the postzygapophyses, and only connected by a thin horizontal lamina of bone (1). (Rauhut 2003).

249): Dorsal vertebrae, neural spine, mediolateral expansion of the dorsal surface: absent (0); present (1). (Makovicky and Sues 1998).

250): Vertebrae, posterior dorsal, sacral and anterior caudal neural spines, ratio between the dorsoventral height and the basal anteroposterior length: less (0); more (1) than 5/2. (Modified from Rauhut 2003).

251): Scapula, ratio between the proximo-distal length and the minimal dorsoventral depth: less than (0); more than (1) six times. (Modified from Rauhut 2003).

252): Scapula, proximodistal length: less than 9/10 of humerus (0); subequal or longer than 9/10 of humerus (1).

253): Scapula, dorsal and ventral margins in lateral/costal view: markedly diverge posteriorly (0); not diverging posteriorly (1).

256): Scapula, acromion, dorsoventral diameter relative to the minimal dorsoventral diameter of the scapular shaft: less (0); more (1) than 4/5.

258): Scapula, acromion, contact between its dorsal half and the coracoid: present (0); absent (1). (Modified from Currie and Carpenter 2000).

259): Scapula, participation to the glenoid: subequal to the coracoid participation (0); wider than the coracoid participation (1). (Sereno 1999).

264): Coracoid, lateral tubercle: absent (0); present (1). (Modified from Holtz 2000).

267): Coracoid, posteroventral process: indistinct (0); distinct (1). (Modified from Holtz 2000; Senter 2010).

268): Coracoid, lateral tubercle, longitudinal extent: short (boss/tubercle) (0); anterodistally elongate (ridge) (1). (Modified from Novas 1997; Benson et al. 2010).

271): Humerus, head, shape in proximal view: ellipsoidal (0); rounded (1). (Modified from Rauhut 2003; O’Connor 2009).

273): Humerus, proximoventral tuberosity, shape: proximodistally short (0); proximodistally elongate (1). (Modified from Rauhut 2003).

275): Humerus, deltopectoral crest, proximodistal length: less than (0); more than (1) 2/5 of the length of the humerus. (Modified from Gauthier 1986).

278): Humerus, deltopectoral crest, maximus anteroposterior diameter: subequal or more (0); less (1) than mid-shaft anteroposterior diameter of the humerus. (Modified from Rauhut 2003; Wilson et al. 2003).

279): Humerus, distal condyles, position: distally placed (0); anteriorly placed (1). (O’Connor 2009).

281): Humerus, proximodistal length: subequal or more (0); less (1) than 3/5 of the length of the femur.

282): Humerus, distal condyles, shape: broadly convex or hemispherical (0); proximodistally depressed, flattened (1). (Modified from Wilson et al. 2003).

283): Humerus, distal epiphysis, mediolateral width: less than (0); more than (1); 3/2 of mid-shaft humeral width.

287): Ulna, olecranon process, proximodistal length: less (0); more (1) than 1/4 of the length of the ulnar shaft.

289): Radio-ulnar distal syndesmosis: absent (0); present (1). (Holtz 2000).

291): Radius, proximodistal length: more than (0); less than (1) 3/5 of the proximodistal length of the humerus.

292): Manual phalanx P1-III, length: more than (0) less than (1) 3/5 of the proximodistal length of manual phalanx P1-II.

294): Distal carpals: proximodistally uncompressed bones with distinct articular surfaces (0); discoidals (proximodistally compressed) without distinct articular surfaces (1). (Modified from Holtz 2000).

295): Distal carpals 1 and 2: distinct (0); fused into a single wide laterodistal carpal capping both mcs I and II (1). (Modified from Rauhut 2003).

296): Mc II, proximodistal length: less (0); more (1) than 2 times the mediolateral width of its distal articular surface. Ordered. (Modified from Rauhut 2003).

297): Mc I, shape in proximal view: broad trapezoid with rounded medial side (0); narrow triangular, with sharp dorsomedial edge (1). (Modified from Rauhut and Xu 2005).

298): Distal carpal 5: present (0); absent or unossified (1).

299): Mc I, proximal surface, medial half contacting the distal carpals: present (0); absent (1). (O’Connor 2009).

300): Mc I, lateral surface, extent of the articulation for Mc II: proximally reduced to the proximal third of the bone (0); distally expanded over the proximal third of the bone (1). (Modified from Rauhut 2003).

301): Mc I, proximodistal length: more (0); less (1) than 3/5 of the length of Mc II.

302): Mc I, minimal mediolateral diameter: more than (0); less than (1) ¼ of the proximodistal diameter of the same mc. (Modified from Rauhut 2003).

303): Mc I, laterodistal condyle: subequal in proximodistal extension (0); proximodistally more elongate (1) than the lateromedial condyle. (Rauhut 2003).

304): Mcs, distal end, extensor pits: poorly developed (0); deep and well developed (1). (Rauhut 2003).

305): Manual phalanx P1-I, lateral surface, proximodorsal process, development: poorly developed (0); pronounced (1). (Modified from Sereno 1999).

306): Manual phalanx P1-I, ventrodistal (flexor) fossa: absent or shallow (0); present and deep (1). (Sereno 1999).

308): Manual phalanx P1-I, proximodistal length: no more than 3/2 (0); more than 3/2 (1) of Mc I length.

309): Manual ungual I, proximodistal length: less than (0); more than (1) manual phalanx P1-I length. The proximodistal length of the unguals is defined as the length of the segment connecting the proximodorsal border and the distal tip, in lateral view.

310): Mc II, medial side in dorsal/ventral view: mediolaterally expanded proximally (0); mediolaterally unexpanded proximally (1). (Rauhut 2003).

312): Manual digit I (P1-I + P2-I), proximodistal length relative to manual digit II (P1-II + P2-II + P3-II): longer (0); shorter (1).

313): Manual phalanx P1-I, proximodistal length: less than 5 times (0); more than 5 times (1) the mediolateral width at midshaft of the same phalanx. (Senter 2010).

314): Manual digit II (P1-II + P2-II + P3-II), proximodistal length: less than (0); more than (1) 4/5 of proximodistal length of the humerus.

316): Manual phalanx P2-II, proximodistally length: subequal or shorter than (0); longer than (1) manual phalanx P1-II.

319): Mc IV, shape in dorsal/ventral view: mediolaterally expanded proximally (0); mediolaterally unexpanded proximally (1).

320): Mc III, shape in proximal view: quadrangular (0); subtriangular, wider ventrally than dorsally (1). (Modified from Rauhut 2003).

321): Mc III, proximal end, mediolateral diameter compared to mid-shaft diameter: wider (0); subequal (1). (Modified from Holtz et al. 2004).

322): Mc III, mediolateral width of mid-shaft: more than (0); less than (1) 3/5 of mc II mid-shaft width. (Modified from Holtz 2000).

323): Mc IV, mediolateral width of mid-shaft of Mc: more than 1/2 (0); subequal or less than 1/2 (1) of mid-shaft diameter of Mc II. (Modified from Holtz et al. 2004).

324): Mc III, proximodistal length: more than (0); less than (1) 3/4 of Mc II.

326): Manual ungual III, proximodistal lenght: longer than 3/4 (0); less than 3/4 (1) of manual ungual II.

328): Manual phalanx P3-III, proximodistal lenght: subequal to or less than (0); longer than (1) the sum of the preceding phalanges. (Modified from Holtz 2000).

329): Mc IV, proximodistal length: more than (0); less than (1) ½ of Mc II length. (Modified from Holtz et al. 2004).

330): Manual digit IV, phalanges: present (0); absent (1). Inapplicable in taxa lacking Mc IV.

331): Mc V: present (0); absent or unossified (1).

332): Manual unguals II and III, transversely expanded proximodorsal lip distinct from rest of dorsal margin: absent (0); present (1). (Modified from Senter 2010).

333): Manual unguals, flexor tubercles, dorsoventral diameter: less (0); more (1) than 1/3 the depth of the proximal articular surface. (Rauhut 2003).

334): Manual unguals II-III, shape in lateral view: straight or slightly curved ventrally (0); distinctly curved ventrally (1).

335): Sternal plates: unfused (0); fused (1).

344): Sacrum, ribs, central articulation, anteroposterior extent: on a single centrum (0); on two centra (1). (Nesbitt 2011; modified from Brusatte et al. 2010).

345): Sacral vertebrae, posterior central keel: absent (0); present (1). (Modified from Holtz 2000; Senter 2010).

347): Sacral vertebrae, pleurocoels: absent (0); present (1).

348): Sacral ribs, lateral surface: slender (0); dorsoventrally hypertrophied, covering almost the entire medial surface of the iliac blades (1). (Modified from Rauhut 2003).

350): Sacral neural spines: unfused (0); fused into a lamina (1). (Holtz 2000).

351): Caudal vertebrae, median and distal centra, mediolateral width of the distal articular surface: less than (0); more than (1); 3/2 of the dorsoventral diameter of the same articular surface. (Modified from Rauhut and Xu 2005).

354): Caudal vertebrae, chevrons, middle elements, anteroventral process: absent (“L-shaped” chevron) (0); present (“T-shaped” chevron) (1). (Modified from Holtz 2000).

355): Caudal vertebrae, chevrons, middle elements, anteroposterior length: less (0); more (1) than 3/2 centrum length. (Modified from Holtz 2000; Zhou and Zhang 2002).

357): Caudal ribs, shape of the anterodistal margin in dorsal/ventral view: unexpanded (0); anteriorly expanded (1). (Modified from Carrano and Sampson 2008).

358): Caudal vertebrae, anterior neural arches, ventral rib laminae: absent (0); present (1).

359): Caudal vertebrae, anterior neural arches, hyposphene: absent (0); present (1).

360): Caudal vertebrae, anterior centra, shape: cylindrical (0); box-like (1). (Rauhut 2003).

361): Caudal vertebrae, distal neural arches, longitudinal groove on the dorsal surface: absent (0); present (1). (Modified from Senter 2010).

362): Caudal vertebrae, anterior centra, longitudinal groove on the ventral surface: absent (0); present (1). (Holtz 2000; Rauhut 2003).

365): Caudal vertebrae, anterior centra, shape of the ventral surface: flattened or slightly convex medio-laterally (0); strongly constricted medio-laterally or keeled (1). (Modified from Rauhut 2003).

367): Caudal vertebrae, anterior and median neural spines, shape of the proximal margin in lateral view: straight (0); having a distinct kink, the dorsal part of the anterior margin more strongly inclined posteriorly than the ventral part (1). (Rauhut 2003).

368): Caudal vertebrae, median neural spines, shape in lateral view: taller than long (0); longer than tall (1).

369): Caudal vertebrae, accessory neural spine (or spur) placed on anterior end of neural arch: absent (0); present, producing a concave dorsal margin of the neural spine (1). (Modified from Rauhut 2003).

372): Caudal vertebrae, centra, pleurocoels: absent (0); present (1). (Holtz 2000; Maryańska et al. 2002).

373): Caudal vertebrae, transition point in tail (transition between the morphologies of the anterior and posterior regions of the tail): gradual (0); localised in a restriced portion of the tail (1). (Modified from Gauthier 1986).

374): Caudal vertebrae, proximal and median ribs, inclination in anterior/posterior view: laterally directed or slightly dorso-laterally directed (0); strongly dorso-laterally directed (1). (Canale et al. 2008).

375): Caudal vertebrae, posterior chevrons, distal forking: absent (0); present (1). (Modified from Senter 2010).

376): Ilium, ischial articulation in adult: unossified (0); ossified (1). (Modified from Tykoski 2005).

377): Ilium, preacetabular process, anteriormost extent: placed posteriorly (0); placed anteriorly (1) to the anteriormost extent of the pubic peduncle of the ilium.

378): Ilium, antero-posterior length: less than 3/5 (0); longer than 3/5 (1) of the proximodistal length of the femur.

380): Ilium, supracetabular shelf covering the anterodorsal margin of the acetabulum: absent (0); present (1). (Modified from Tykoski 2005).

381): Ilium, lateral horizontal supracetabular crest, development: slightly developed dorsal lip of the acetabulum (0); prominent (1). (Modified from Holtz 2000).

382): Ilium, lateral vertical crest dorsal to the acetabulum: absent (0); present (1). (Rauhut 2003).

383): Ilium, supracetabular crest, posteriormost extent: placed anteriorly (0); dorsally (1) to the ischial peduncle of the ilium.

385): Ilium, preacetabular process, ventralmost extent: dorsal to (0); at the same level of (1) the iliopubic facet.

387): Ilium, preacetabular process, anterodorsal concavity in lateral view: absent (0); present (1). (Modified from Rauhut 2003).

388): Ilium, pubic peduncle, shape in lateral view: straight to convex (0); concave, with an anterior lip at its ventral margin (1). (Modified from Rauhut 2003).

389): Ilium, pubic peduncle, ventral surface: single (0); double, with a pronounced kink between the anterior surface facing almost entirely anteriorly and the posterior surface facing ventrally (1). (Modified from Carrano and Sampson 2008; Rauhut 2003).

390): Ilium, pubic peduncle, anteroposterior diameter: less than (0); subequal to or more than (1) the anteroposterior diameter of the acetabulum.

391): Ilium, ischial facet, anteroposterior diameter relative to the anteroposterior length of the pubic facet: subequal to longer (0); shorter (1).

392): Ilium, border formed by the anterior margin of the pubic peduncle and the posteroventral margin of the preacetabular process, shape in lateral view: wide concavity, describing an arch broader than 35° (0); anteroventrally oriented cleft, describing an arch narrower than 35° (1).

394): Ilium, pubic peduncle, dorsoventral depth: subequal or less than (0); deeper than (1) the dorsoventral depth of the ischial peduncle.

396): Ilium, pubic peduncle, ventral surface, anteroposterior length: less than (0); more than (1) 2 times its mediolateral width. (Modified from Rauhut 2003). The same condition may be inferred from the dorsal surface of the iliac peduncle of the pubis in specimen lacking the pubic peduncle of the ilium.

397): Ilium, postacetabular process, dorsal margin, orientation in lateral view when the sacral column is horizontal: subhorizontal, distinct from posterior margin (0); posteroventrally directed, confluent with posterior margin (1). (Modified from Carrano et al., 2002; Makovicky et al., 2005).

399): Ilium, preacetabular processes, orientation in dorsal view: subparallel (0); anteromedially directed, contacting the neural spines of the anterior sacrals (1). (Modified from Holtz 2000; Zanno 2010).

401): Ilium, postacetabular process, ventral margin, orientation in lateral view: posterodorsally to subhorizontally (0); posteroventrally (1) directed.

402): Ilium, postacetabular process, shape in dorsal view: posteriorly directed (0); posterolaterally directed (1). (Senter 2010).

404): Ilium, preacetabular process, fossa on the lateroventral margin: very shallow, indistinguible from the rest of the blade (0); present as a marked concavity between the anterior margin of the pubic peduncle of the ilium and the ventral margin of the preacetabular blade (1). (Modified from Holtz 2000).

405): Ilium, postacetabular process, medioventral shelf, anterior half, lateral exposure: absent (0); present (1). (Modified from Carrano et al. 2002).

406): Pubis, proximoanterior margin, shape in lateral view: straight, absence of marked anterior espansion of the iliac peduncle relative to the shaft (0); markedly convex proximally, iliac peduncle expanded anteriorly (1).

407): Pubis, shaft, shape in lateral view: straight to anteriorly concave (0); posteriorly concave (1). (Holtz 2000).

409): Pubis, ventral foot, posterior process: absent (0); present (1). (Modified from Holtz 2000).

410): Pubis, ventral surface, shape in ventral view: subtriangular, anteriorly broader (0); quadrangular, with subparallel lateral margins (1). (Modified from Rauhut; 2003).

411): Pubis, ventral foot, anterior process: absent (0); present (1). (Modified from Holtz 2000).

412): Pubis, ischial peduncle, obturator perforation: present as foramen or notch (0); solid bone with no distinct perforation (1). (Modified from Holtz 2000).

413): Pubis, ischial peduncle, obturator perforations, number: zero or one (0); two (1). (Modified from Rowe and Gauthier 1990). (Tykoski 2005).

415): Pubis, ventral contact between the hemipubic bones (pubic symphysis) in anterior view: present (0); absent (1).

416): Pubis, pubic apron, median perforation: absent (0); present as a fenestra (1). (Modified from Holtz 2000; Rauhut 2003; Makovicky et al. 2005).

420): Ischium, shape in lateral/medial view: straight (0); posterodorsally concave at the level of the obturator process (1). (Modified from Senter 2010).

422): Ischium, proximodorsal process: absent (0); present (1). (Modified from Forster et al. 1998).

423): Ischium, mediodorsal process: absent (0); present (1). (Modified from O’Connor 2009).

424): Ischium, scar on the proximolateral surface: slightly developed (0); prominent, crescentic (1) (Holtz 2000).

425): Ischium, ventral half, cross section: laminar, strongly mediolaterally compressed (0); robust, rod-like (1).

426): Ischium, obturator process/lamina, distal margin, cleft/concavity: absent (0); present (1). (Rauhut 2003).

427): Ischium, ventral expansion of shaft (ischial foot): absent (0); present (1). (Modified from Holtz 1994).

428): Ischium, ventral symphysis: present (0); absent or unossified (1).

431): Femur, head and neck, proximodistal axis in proximal view, inclination relative to mediolateral axis of distal end: anteromedially directed (angle of more than 30°) (0); slightly anteromedially to medially directed (angle less than 30°) (1). (Modified from Carrano and Sampson 2008; Rauhut 2003).

432): Femur, head and neck, proximodistal axis, inclination relative to shaft main axis, angle in anterior/posterior view: no more than (0); more than (1) 90°. (Holtz 2000).

433): Femur, accessory trochanter: absent (0); present as a anterodistal process of the anterior trochanter (1). (Sereno 1999).

435): Femur, trochanteric shelf, development: poorly developed anterolateral crest (0); prominent, shelf-like (1). (Modified from Tykoski 2005).

436): Femur, “posterior” trochanter: absent (0); present as a posterolateral mound-like eminence (1). (Hutchinson 2002).

437): Femur, fourth trochanter, shape: low rugosity/scar (0); sharp flange (1). (Modified from Sereno 1999; Langer and Benton 2006).

438): Femur, distal end, medial condyle, shape: distally flattened (0); distally rounded (1). (Rauhut 2003).

439): Femur, distal end, anterior margin in distal view: flat to convex (0); concave (1). (Modified from Currie and Carpenter 2000; Holtz 2000).

440): Femur, distal end, anterior intercondylar sulcus, depth: broad and shallow (0); deep and narrow (1).

441): Femur, medial condyle, anteroposterior diameter in distal view: less than (0); subequal or more than (1) the mediolateral diameter of the distal articular surface of the femur.

444): Femur, posteromedial margin, elliptical scar for the inserton of the M. caudifemoralis longus: slightly developed (0); marked (1). (Modified from Brusatte et al. 2010).

445): Femur, posterodistal fossa in distal view, cruciate ligament: unossified (0); ossified (1). (Currie and Carpenter 2000).

446): Femur, anterior trochanter, shape in lateral view: cone-like to finger-like (0); flange-like (1).

447): Tibia, proximal end, elongation in lateral/medial view: no more (0); more (1) than twice the minimal anteroposterior diameter of the shaft.

448): Fibula, proximomedial surface, shape: flat or slightly concave (0); deeply concave (1). (Modified from Rauhut 2003).

449): Tibia, fibular condyle, posteriormost estent in proximal view: anteriorly to (0); at the same level of (1); the posterior margin of the medial condyle.

451): Tibia, fibular condyle in proximal view, anterior process: absent (0); present (1). (Brusatte et al. 2010).

452): Tibia, fibular crest: absent (0); present (1).

454): Tibia, distal end, posterolateral process, calcaneal facet/contact: absent (0); present (1). (Modified from Sereno 1999; Holtz 2000).

455): Tibia, distal end, mediolateral diameter: subequal or less (0); more (1) than the anteroposterior diameter. (Modified from Rauhut 2003).

457): Tibia, distal end, fossa/slot for the astragalar ascending process: absent (0); present (1). (Modified from Rauhut 2003).

461): Tibia, distal end, anteroposterior length of medial margin: subequal to (0); longer than (1) the anteroposterior length of the lateral margin. (Langer and Benton 2006).

462): Astragalus, anterior surface proximal to condyles, transversal groove: absent (0); present (1). (Holtz 2000).

463): Astragalus, posteromedial process (and corresponding fossa on the posterodistal margin of the tibia): absent (0); present (1).

465): Astragalus, distal condyles, position: completely distally or slightly anterodistally placed (0); mostly anterodistally placed (1). (Modified from Holtz 2000).

466): Tibia, proximal end, posterior cleft between the proximal condyles, development: indistinct (0); deep (1). (Rauhut 2003).

467): Fibula, relationships with the astragalar-tibial complex in adult: unfused or loosely appressed (0); tightly appressed or fused (1).

468): Astragalus, ascending process, mediolateral diameter of the base: no more than (0); more than (1) 1/2 of mediolateral diameter of the astragalar body. (Modified from Rauhut 2003).

469): Astragalus, ascending process, proximodistal diameter: less than (0); more than (1) the mediolateral diameter of the astragalar body. Ordered. (Modified from Rauhut 2003).

473): Calcaneum, mediolateral diameter: more than 1/3 (0); less than 1/3 (1) of the mediolateral diameter of the astragalus.

475): Distal tarsals, relationships with the proximal end of mts: unfused (0); fused (1).

476): Mts II-IV, shafts, proximal half, relationships: unfused (0); fused (1). (O’Connor 2009).

477): Mt I, proximodistal length: more than 2/5 (0); less than 2/5 (1) of the proximodistal length of Mt II.

478): Mt III, proximodistal length: less (0); more (1) than 1/2 of tibial lenght.

481): Mt II, distal articular surface, shape: flat or sligtly concave (0); markedly concave, with distinct extensor groove (1). (Senter 2010).

482): Mt III, shaft, distal half, posterior margin, shape: broad to rounded (0); mediolaterally constricted/sharp (1). (Holtz 2000).

483): Mt III, shaft, proximal half, mediolateral compression relative to mid-shaft width: absent (0); present (1). (Holtz 1995).

484): Mt III, proximal surface, anteroposterior diameter: no more than 5/4 (0); more than 5/4 (1) of the anteroposterior diameter of the proximal surface of Mt II.

485): Mt III, proximal surface, development: subequal to or more than (0); less than (0); those of the proximal surfaces of both mts II and IV (1).

486): Mt III, proximal surface, anterior margin, position relative to the anterior margins of the proximal surfaces of both mts II and IV: at the same level (0); more posteriorly/plantarly (1). (Modified from O’Connor 2009; Holtz 2000).

487): Mt III, distal articular surface, shape: flat or slightly concave (0); markedly concave, with distinct extensor groove (1). (Senter 2010).

488): Mt III, distal end, mediolateral diameter: subequal to or more than (0); less than (1) the mediolateral diameter of the distal end of Mt II. (O’Connor 2009).

489): Mt II, shaft excluding distal fourth, shape in anterior/posterior view: sygmoidal, mediodistally concave (0); straight (1).

490): Mt IV, shape in anterior/posterior view: sigmoidal, laterodistally concave (0); straight (1).

491): Mt IV, proximal end, medial margin, shape: flat to convex (0); concave (1).

493): Mt IV, proximal end in anterior/posterior view, lateral projection clearly distinct from shaft margin: present (0); absent (1). (Modified from Senter 2010).

495): Mt IV, distal end, mediolateral diameter: subequal to or more than (0) less than (1) 3/5 of the mediolateral diameter of the distal end of Mt III. (Modified from Wilson et al. 2003).

497): Mt V, proximodistal length: more than (0); less than (1) 2/5 of the proximodistal length of Mt III.

499): Pedal phalanx P1-I, proximodistal length: more than 3/4 (0); no more than 3/4 (1) of the proximodistal length of pedal phalanx P1-III. (Modified from Sereno 1999).

500): Mt I, proximal end, position: at the same level (0); distally to (1) the proximal end of Mt II.

501): Pedal phalanx P1-II, proximodistal length: no more than 3 times (0); longer than 3 times (1) the proximodistal length of its distal trochlear eminence. (Senter 2010).

502): Pedal phalanx P2-II, proximodistal length: more than 2 times (0); no more than 2 times (1) the proximodistal length of its distal trochlear eminence. (Senter 2010).

506): Pedal phalanx P1-IV, proximodistal length: subequal to or more than (0); less than (1) the proximodistal length of pedal phalanx P1-II. (Senter 2010).

507): Pedal digit V, phalanges: present (0); absent or unossified (1).

508): Pedal unguals II-IV, lateral vascular grooves, shape: simple, unforked (0); proximally forked, producing a pair of distally converging furrows (1). (Modified from Carrano and Sampson 2008).

518): Manual unguals, flexor tubercles, position of proximal margin: close to (0); distal to (1) the ventral margin of the proximal surface of the ungual. (Sereno 1999).

519): Frontals, paired bones, posteromedial margin, shape in dorsal/ventral view: straight or posteriorly convex (0); notched and markedly concave at mid-length (1).

521): Pubis, ischial peduncle, dorsoventral diameter of the distal articulation: subequal to or more than (0); less than (1) 3/2 of the minimal anteroposterior diameter of the pubic shaft.

522): Skull, anterior bones (premaxilla, maxilla and dentary), medial surface, texture: smooth (0); skulptured and furrowed (1). (Carrano and Sampson 2008).

523): Manual phalanx P1-I, proximoventral margin, paired flexor processes: absent (0); present (1). (Sereno 1999).

525): Postorbital, anterodorsal process, position: in line with the posterior process (0); strongly upturned, both processes describe a concave postorbital border of the supratemporal fossa (1). (Senter 2010).

526): Articular, pendant medial process: absent (0); present (1) (Sereno 1999).

527): Mt III, proximal end, posterior border, width relative to anterior margin width: much wider (0); comparable or reduced (1).

528): Nasals, posterior end, minimal mediolateral diameter: subequal to or more than (0); less than (1); the mediolateral diameter of the anterior half of the nasal. (Modified from Holtz et al. 2004).

529): Pubis, foot, proximodistal diameter: less than (0); more than (1) 1/4 of the proximodistal length of the pubis.

530): Astragalus, ascending process, fibular articular facet: absent (0); present (1).

531): Mc II, mediolateral diameter at mid-shaft: more than (0); less than (1) 2/3 of the mediolateral diameter mid-shaft of Mc I.

532): Maxilla, maxillary fenestra, dorsal margin, position: at midheight (0); in the anterodorsal corner (1) of the antorbital fossa. (Senter et al. 2004).

533): Lacrimal, posterodorsal corner, dorsal margin, horn: absent (0); present (1).

534): Basisphenoid, internal pneumatic recesses, development: poorly developed (0); extensive, well developed (1). (Senter 2010).

535): Basioccipital, subcondylar recess: absent (0); present (1).

536): Maxilla, lateral surface, neurovascular foramina: present (0); absent (1).

537): Skull, dorsoventral diameter of the snout at the level of the posterior margin of the external naris: more than (0); less than (1) 2/5 of the dorsoventral diameter of the orbit.

538): Supraoccipital, posterodorsal margin, posteriormost extent: anteriorly to or at the same level of (0); posteriorly to (1) the foramen magnum. (Wilson et al. 2003).

539): Humerus, shape in lateral/medial view: straight (0); sigmoid, with the proximal surface inflected posteriorly (1). (Modified from Holtz 2000; Rauhut 2003; Carrano et al. 2011).

542): Ischium, obturator process, elongation in lateral/medial view: more elongate proximodistally than anteroposteriorly (0); more elongate anteroposteriorly than proximodistally (1). (Makovicky et al. 2005).

545): Mt II, trochlea, distal end, position relative to Mt III when articulated: at the level of (0); more proximal than (1) the trochlea of Mt III.

546): Nasal, posterolateral process: absent (0); present (1). (Wilson et al. 2003; Brusatte et al. 2010).

547): Mc II, proximodistal length: no more than (0); more than (1) 1/3 of the proximodistal length of the humerus.

548): Pedal ungual I, size relative to pedal unguals III or IV: smaller (0); subequal or larger (1). Pedal ungual II is excluded from these character conditions to avoid redundancy with other character statements.

549): Jugal, contribution to the antorbital fossa, shape in lateral view: fossa absent or poorly developed (0); half-crescentic with a distinct posterior rim (Rauhut 2003).

551): Dentary, anterodorsal corner, angle between the anterior and the dorsal margins in lateral view: more than (0); less than (1) 65°. (Modified from Senter 2010).

553): Maxilla, interfenestral bar (between the antorbital and the maxillary fenestrae), internal cavitation: absent (0); present (1).

554): Supratemporal fossa, angle between the anteroposterior axis and the anteroposterior axis of skull: subparallel (angle < 15°) (0); rostroventrally inclined (angle > 15°) (1). (Modified from Coria and Currie 2002).

555): Cervical vertebrae, centra, anterior surface, shape in anterior view: elliptical or subcircular, dorsally convex (0); kidney-shaped, dorsally concave (1). (Modified from Holtz 2000; Rauhut 2003).

556): Fibula, insertion of the M. ileofibularis, shape: simple process (0); transversely paired processes (1). (Holtz et al. 2004).

558): Ulna, olecranon, shape in proximal view: mediolaterally broad and anteroposteriorly low (0); mediolaterally compressed and blade-like (1). (Smith et al. 2008; Benson et al. 2010).

559): Scapula, acromion, inclination of the posterior margin relative to shaft: steeply inclined dorsally (0); gently sloping (1).

560): Mt IV, cross section at mid-shaft, shape: deeper than wide, transversely compressed (0); as wide as deep or wider, uncompressed (1). (Modified from Senter 2010).

561): Tibia, laterodistal end, distalmost extent: proximally to or at the same level of the distal extent of the mediodistal end (0); distally to the distal extent of the mediodistal end (1).

562): Caudal vertebrae, chevrons, anteroproximal process: absent (0); present (1). (Rauhut 2003).

565): Maxilla, antorbital diverticulum connecting the external naris and the antorbital fossa: absent (0); present (1).

569): Paroccipital process, shape: elongate and slender, with dorsal and ventral edges nearly parallel (0); short, deep with convex distal end (1). (Senter 2010).

571): Lacrimal, ventral process, participation to the posterior margin of the antorbital fossa: present (0); absent (1).

573): Femur, distal end, lateral condyle, area placed lateral to the ectocondylar tuber, mediolateral diameter in distal view: less than 1/3 (0); more than 1/3 (1) of the mediolateral width of the lateral condyle of the femur.

574): Lacrimal, dorsal surface: less than (0); more than (1) the prefrontal dorsal surface (1).

575): Tarsometatarsus, proximal surface, plantar expansion (hypotarsus): absent (0); present (1). (Modified from O’Connor 2009).

577): Pubis, foot, ventral margin, shape in lateral view: straight or slightly convex (0); broadly convex (1).

578): Tibia, anterodistal end, proximodistally elongate medial crest: absent (0); present (1). (Modified from Rauhut 2003).

580): Tibiotarsus, complete fusion in adult: absent, proximal tarsals and tibia unfused, sutures clearly visible (0); present, astragalocalcaneum fused to the tibia (1).

582): Skull, rostrum, mediolateral constriction at the level of the premaxilla-maxilla articulation: absent (0); present (1). (Sereno 1999; Rauhut 2003).

583): Distal tarsal 4, lateroposterior margin, shape in proximal/distal view: convex or slightly concave (0); broadly concave (1).

584): Radius, proximoposterior process, development: poorly developed (0); prominent (1).

585): Ilium, dorsoventral diameter at the level of the ventral margin of the pubic peduncle: subequal to or more than (0); less than 3/5 of the anteroposterior length of the ilium (1).

586): Premaxilla, buccal margin in ventral view, orientation: more anteroposteriorly than mediolaterally (0); more mediolaterally than anteroposteriorly (1). (Modified from Holtz et al. 2004).

587): Axis, neural spine, anterodorsal margin, shape in lateral view: broadly concave or straight (0); broadly convex (1).

588): Humerus, proximoventral tuberosity, development: moderately developed, posteriorly projected (0); hypertrophied, posteromedially projected (1). (Modified from Zanno 2010).

589): Premaxilla, palatal shelf, incisive foramen at mid-length of the medial articulation: absent (0); present (1).

592): Ilium, brevis fossa, lateral and medial margins, orientation in ventral view and development of fossa: subparallel, narrow fossa (0); posteriorly diverging, expanded fossa (1). (Modified from Holtz 2000; Rauhut 2003).

593): Surangular, preglenoid process, development: poorly developed (0); prominent (1).

594): Anterior trochanter, apex, proximalmost extent when the femur is vertically oriented: distally to (0); at the same level to (1) the dorsal margin of femoral head. (Modified from Holtz, 2000).

598): Premaxilla, ventral process, posterior bifurcation: absent (0); present (1). (Rauhut 2003).

599): Maxillary/dentary teeth, crowns, labiolingual diameter relative to mesiodistal diameter at mid-crown: less than (0); more than (1) 3/5. (Modified from Currie and Carpenter 2000; Holtz 2001).

600): Premaxillary teeth, lingual surface, apicobasally elongate median ridge: absent (0); present (1). (Brusatte et al. 2010).

601): Mt II, mid-shaft, width: more (0); less (1) than 1/2 distal end width.

606): Ilium, posterior margin, posteroventral process (and brevis shelf) projected posteriorly beyond level of mid-height of posterior margin: absent (0); present (1). (Modified from Makovicky et al. 2005).

608): Humerus, deltopectoral crest, distal margin, shape formed with humeral shaft: broad arch (0); narrow margin, describing an angle close to 90° (1).

609): Premaxillary and dentary teeth, anteriormost teeth, apicobasal axis (when fully erupted), inclination: sub-perpendicular to the anteroposterior axis of the skull (0); mesioapically inclined, procumbent (1).

610): Manual unguals, proximal surface, height-width ratio: less than (0); more than (1) 3/2.

611): Cervical vertebrae, epipophyses, dorsalmost extent: ventrally to (0); at the same level or dorsally to (1) the dorsal surface of the neural spine.

612): Mt III, proximal view, mediolateral constriction at mid-length of its anteroposterior axis: absent or poorly developed (0); present and marked (1). (Modified from Holtz 2000).

613): Ilium, preacetabular process, ventralmost extent, position in lateral view: closer to the anterior margin of the ilium (0); close to the mid-point of the anteroposterior axis of the preacetabular process (1).

614): Maxilla, facet of the nasal, inclination: facing laterally (0); facing ventrally (1). (Wilson et al. 2003).

615): Lacrimal, nasolacrimal duct, position: leading through the body of the ventral process (0); passing lateral to the ventral process (1). (Modified from Rauhut 2003).

616): Frontal, dorsal surface, sagittal crest: absent (0); present at least on the posterior half (1).

619): Upper tooth-row, posterior extent relative to dentary tooth-row: at the same level or posterior (0); anterior (1). (Sereno 1999).

620): Manual ungual III: present (0); absent (1).

626): Caudal vertebrae, anterior and middle neural arches, prezygocostal lamina: absent (0); present (1).

627): Ischium, obturator foramen, ossification of the ventral border: complete, connecting the pubic peduncle of the ischium with the obturator lamina (0); open notch (1).

628): Ilium, ischial peduncle, distal end, shape in lateral view: broad and flat articular surface (0); subtriangular in lateral view, terminates in a reduced and convex articular surface (1). (Modified from Rauhut 2003).

629): Caudal vertebrae, posterior prezygapophyses, overlapping of the preceding centrum: no more than (0); more than (1) 30% of the length of the preceding centrum. (Modified from Holtz 2000).

630): Pubis, foot, shape in anterior/posterior view: mediolaterally unexpanded (0); mediolaterally expanded, wider than the mediolateral width of the pubis just proximally to the pubic foot (1).

632): Occipital condyle, shape in posterior (occipital) view: rounded, dorsally convex (0); kidney-shaped, slightly concave dorsally (1).

633): Exoccipital, participation to the dorsal margin of the foramen magnum, mediolateral diameter: less than 1/2 (0); more than 1/2 (1) of the mediolateral diameter of the foramen magnum. (Modified from Holtz 2000).

634): Mc II, proximomedial margin, shelf overlapping Mc I: absent (0); present (1).

635): Nasal, lateral crest, posterior processes posterodorsally directed: absent (0); present (1).

636): Femur, distal end, medial margin, proximodistally elongate crest: absent (0); present (1). (Modified from Carrano and Sampson 2008).

637): Frontal, dorsal surface, anteromedial eminence: absent (0); present (1). (Modified from Carrano and Sampson 2008).

638): Cervical vertebrae, epipophyses, anterior process: absent (0); present (1). (Coria and Salgado 2000).

639): Caudal vertebrae, anterior chevrons, bridge of bone closing dorsally the hemal canal: absent (0); present (1). (Modified from Holtz 2000).

640): Humerus, shaft, posterior tubercle: absent (0); present (1). (Modified from Zanno 2010).

641): Manual phalanges, ligament pits, development: strongly developed (0); weakly developed (1). (Zanno 2010).

643): Prearticular, medial process: absent (0); present (1). (Modified from Norell et al. 2001; Carr 2005).

644): Quadrate, mandibular condyles, position: placed distally (0); placed anterodistally (1).

645): Lacrimal, anterodorsal process, dorsoventral depth: subequal to (0); less than (1) the minimal anteroposterior length of the ventral process (0). (Modified from Sereno 1999).

646): Humerus, shaft, proximodistally oriented posterior sulcus: absent (0); present (1).

647): Cervical neural arches, postzygapophyses, anterolateral surface, foramina: absent (0); present (1). (Allain et al. 2007).

648): Scapula, dorsal margin, costolateral expansion compared to the ventral margin: absent (0); present (1).

649): Squamosal, ventral (precotyloid) process, main axis, inclination in lateral view: posteroventrally directed (0); anteroventrally directed (1).

650): Mt I, proximal end: broad and unconstricted (0); constricted, mediolaterally narrower than the distal half of the bone (1).

651): Dentary, anterior half, medial paradental sulcus separing the interdental septa from the lingual bar: absent (0); present (1).

652): Maxilla, ascending process, lateral pneumatic recesses: absent (0); present (1).

658): Pedal unguals III and IV, shape in lateral view: ventrally curved (0); straigth (1). (Modified from Senter 2010).

659): Mc IV: present (0); absent or unossified (1).

660): Dorsal vertebrae, accessory centrodiapophyseal lamina: absent (0); present (1). (Benson et al. 2010).

662): Lacrimal, angle between the anterodorsal and the ventral rami in lateral view: more than (0); less than (1) 60°. (Sereno 1999).

663): Ischio-pubic medioventral shelves, development: broad and widely contacting medially (pelvic foramen reduced) (0); reduced (wide pelvic fenestra) (1). (Modified from Sereno 1999).

664): Manual ungual I, proximodistal length: less than (0); more than (1) 2/5 of the proximodistal length of the humerus.

665): Caudal vertebrae, median neural spines, dorsoventral diameter: subequal to or less than (0); more than (1) the sum of the dorsoventral diameters of centrum and neural arch.

668): Presacral vertebrae, anterior surface, peduncular fossae placed laterally to the neural canal: absent (0); present (1).

670): Presacral vertebrae, neural arch, dorsal surface, lateral fossae: absent (0); present (1).

671): Manual unguals, ventral surface, proximal half, transversally expanded fossa/e: absent (0); present (1).

676): Mt III, distal end, posterior view, triangular/tonguelike raised processes just proximal to articular surface: absent, distinct intercondylar sulcus present (0); present (1). (Modified from Currie and Dong 2001).

677): Anterior presacral centra, posterior half of centrum, pneumatic recess, development: simple fossa without rim (0); invaginated fossa with a distinct rim (1). (Wilson et al. 2003).

678): Jugal, medial surface, recess placed at the level of the postorbital bar: absent (0); present (1). (Currie and Carpenter 2000; Rauhut 2003; modified from Senter 2010).

681): Tibia, lateral surface, relationships between fibular condyle and cnemial crest: confluent, lateral surface flat to convex (0); condyle offset from the crest by a fossa (1). (Modified from Rauhut 2003; Wilson et al. 2003).

683): Basipterygoid processes, inclination: anteroventrally directed (0); lateroventrally directed (1). (Senter 2010).

685): Tibia, proximal surface, anteroposterior diameter: more (0); less (1) than 9/5 of the mediolateral diameter of the same surface.

686): Mc I, shape in extensor/flexor view: straight (0); mediodistally curved (1).

690): Pubis, proximal end, origin of the M. ambiens, development: present as a scar (0); present as a prominent tuberosity (1).

691): Ischium, minimal anteroposterior diameter at mid-length (excluded the obturator region and the distal tip): subequal to (0); less than (1) the minimal anteroposterior diameter of the pubis at mid-shaft. (Holtz et al. 2004).

695): Premaxilla, region anterodorsal to narial fossa, slot-shaped foramen: absent (0); present (1). (Yates 2006).

696): Articular, erect, tab-like dorsal process, immediately posterior to the opening of the chorda tympanic foramen: absent (0); present (1). (Modified from Yates 2006).

697): Premaxilla, subnarial process, slenderness: shorter than (0); longer than (1) 4 times its proximal depth.

698): Maxilla, antorbital fossa, anterior margin, shape in lateral view: broadly rounded (0); squared, with anteroventrally acute and anterodorsally obtuse corners (1). (Rauhut 2003; Senter 2011).

699): Articular, dorsal surface, attachment area for the M. depressor mandibulae, shape: transversely convex (0); transversely concave (1). (Yates 2006).

700): Axis, neural spine, anterior tip, position: anteriorly to (0); at the same level or posterior to (1) the prezygapophyses. (Tykoski 2005).

705): Astragalus, ascending process, anterolateral margin, groove converging with astragalar base: absent (0); present (1). (Ezcurra and Novas 2007).

707): Maxilla, anteromedial processes, development: short and deep, with little lateral exposure (0); long and low, laterally exposed (1). (Modified from Sereno 1999).

708): Astragalus, ascending process, shape: anteroposteriorly deeper than mediolaterally wide (0); mediolaterally wider than antero-posteriorly deep (1). (Yates 2006)

709): Premaxilla, narial fossa: absent (0); present (1). (Modified from Langer and Benton 2006).

711): Maxilla, anterior process, promaxillary sinus, extension into process: absent (0); present (1) (Sereno 1999).

712): Articular, mandibular glenoid, shape in dorsal view: longer than wide (0); mediolaterally expanded (1). (Rauhut 2003).

716): Basisphenoid, ventral recess, shape: single (0); divided into two small, circular foramina by a thin bar of bone (1) (Senter 2010).

717): Premaxilla, participation to the nasal crest: absent (0); present (1). (Modified from Senter 2010).

720): Maxillary/dentary teeth, interdenticular sulci (blood grooves) in serrations, development: reduced to denticle margin (0); elongate (1).

721): Maxillary/dentary teeth, area adjacent to the marginal carinae, shape: slightly to strongly mesiodistally convex, especially at the mesial carina (0); flat or even slightly concave area adjacent to the marginal carinae (1).

722): Maxilla/dentary, alveoli, shape in apical view: elliptical or suboval (0); quadrangular (1). (Wilson et al. 2003).

723): Dentary, Meckelian groove, mediolateral depth: marked (0); moderate (1). (Modified from Senter 2010).

724): Ilium, supracetabular crest, shape in dorsal/ventral view: semicircular (0); quadrangular (1). (Holtz et al. 2004).

725): Postacetabular process, posterodorsal margin, mediolateral thickness: comparable to the rest of the ilium (0); margin thickened when compared to preacetabular margin (1).

726): Manual phalanx P1-II, proximodistal length: less than 5/2 (0); more than 5/2 (1) of the mediolateral width at mid-shaft of the same phalanx.

728): Humerus, head, shape: expanded more lateromedially than proximodistally (0); proximally inflated (1).

729): Humerus, lateral tuberosity, position: proximally to the medial tuberosity (0); at the same level or distally to the medial tuberosity (1). (Modified from Wilson et al. 2003).

731): Maxilla, anteroventral margin, dorsomedial curvature: absent (0); present (1). (Tykoski 2005).

732): Maxilla, first alveolus, inclination: opens ventrally (0); opens anteroventrally (1). (Tykoski 2005).

733): Nasal, paired laterodorsal crests: absent (0); present (1). (Modified from Holtz 2000).

734): Lacrimal, posterodorsal horn, development in adult: low rugosity (0); prominent, taller than long (1).

739): Cervical vertebrae, anterior neural arches, posterior surface, shape: smooth (0); with an excavation on the posterolateral surface (1). (Langer and Benton 2006).

740): Tibia, distal end, anteromedial corner, angle: subequal or more than (0); less than (1) 90°. (Langer and Benton 2006).

741): Fibula, mid-shaft, mediolateral width: more than 1/2 (0); less than 1/2 (1) of mid-shaft width of the tibia. (Modified from Gauthier 1986).

743): Femur, head, shape in proximal view: oval in contour (0); subtriangular (1). (Ezcurra and Novas 2007).

744): Femur, proximal surface, transversely extended groove: absent (0); present (1). (Ezcurra and Novas 2007).

746): Femur, head, posterior surface, oblique ligament groove, development: very shallow (0); present and deep, bound medially by a posterior lip (1). (Modified from Rauhut 2003).

747): Presacral vertebrae, neural canal, shape: small rounded (0); large oval (taller than 1/3 of centrum depth) (1). (Modified from O’Connor 2009).

749): Manual ungual I, transversely expanded proximodorsal lip: absent (0); present (1). (Senter 2010).

750): Premaxilla, nasal process, contribution to the margin of the external naris: more than (0); less than 1/2 (1) of the anterodorsal border of the external naris. (Modified from Holtz 2000).

751): Maxilla, anterior process, anterolateral margin, tab-like process: absent (0); present (1).

752): Premaxilla, subnarial process, length: subequal to or more than (0); less than (1) the length of the buccal margin of the premaxilla. (Tykoski 2005).

754): Maxilla, anteromedial process, medial surface: smooth (0); bears longitudinal ridges (1). (Sereno 1999).

755): Frontal-parietal, dorsal contact area, medial fossa in depression: absent (0); present (1). (Tykoski 2005).

756): Lacrimal, ventral process, lateral lamina, anterior margin, shape and relationships with the medial lamina: straight, placed posteriorly to medial lamina (0); sinuous, protrudes anteriorly beyond medial lamina (1). (Tykoski 2005).

757): Basisphenoid, transverse intertuberal lamina, shape: simple wall (0); bears small median spur that projects anteriorly along the roof of basisphenoidal recess (1). (Tykoski 2005).

758): Dentary, anterior tip, dorsal edge: continuous with mid-dentary (0); is raised conspicuously relative to middle and posterior parts of dentary (1). (Sereno 1999).

760): Dorsal vertebrae, transverse processes, anteroposterior expansion of base: narrow (0); broad, extending to lateral margin of prezygapophysis (1) (Tykoski 2005).

761): Humerus, shaft torsion, angle between the trasverse axes of proximal and distal ends when viewed proximally/distally: subequal to or less than (0); more than (1) 25°. (Modified from Holtz 2000).

763): Pubis, mid-shaft, mediolateral width: more than 1/4 (0); less than 1/4 (1) of proximoposterior shaft length. (Tykoski 2005).

764): Ischium, antitrochanter, development: small, indistinct (0); large and protrudes anterolaterally into acetabulum, giving 'notched' profile to posteroventral margin of acetabulum (1). (Tykoski 2005; Sereno 1999).

765): Femur, posterodistal (popliteal) fossa in adults, infrapopliteal ridge between medial (=tibial) distal condyle and tibiofibular crest: absent (0); present (1). (Tykoski 2005).

766): Fibula, proximomedial surface, oblique (posteroproximal to anterodistal) ridge that overlaps proximal part of medial fibular groove: absent (0); present. (Tykoski 2005).

767): Lacrimal, posteroventral process, development: short, indistint (0); elongate (1).

768): Fronto-parietal, transversely oriented crest: absent (0); present (1).

769): Postorbital, participation to the supratemporal fossa: wide (0); reduced to the margin of the fossa (1). (Sereno 1999).

770): Squamosal, otic incisure, shape in lateral view: broad and posteriorly directed (0); "inverted-U" shaped and posteroventrally directed (1).

771): Quadrate, distal condyles, helical groove, development: deep and distinct (0); very low and shallow (1).

772): Humerus, distal condyles, intercondylar groove: wider (0); narrower (1) than 1/2 of the mediolateral width of the lateral condyle. (Zanno 2010).

773): Jugal, suborbital process, lateral crest: absent (0); present (1).

774): Maxilla, maxillary fenestra, ventral margin, position: dorsally to (0); at the same level of or ventrally to (1) the ventral margin of the antorbital fenestra. (Brusatte et al. 2010).

775): Ischium, shaft, anteroventral surface, low longitudinal crest placed distally to the obturator process: absent (0); present (1).

778): Pubis, dorsal part of shaft, cross-section, proportion: as deep as wide, or deeper (0); wider than deep (1). (Modified from Senter 2007; O’Connor 2009).

780): Jugal, participation to the posterior border of the orbit: present (0); excluded by the postorbital (1).

782): Cervical vertebrae, anterior centra, posteriormost extent: at the same level or anteriorly than (0); extending posteriorly (1) the posterior extent of the neural arch. (Senter 2010).

785): Premaxilla, nasal processes, distal end, direction in dorsal view: laterally directed, diverging (0); medially directed, appressed (1). (Brusatte et al. 2010).

786): Humerus, shaft distal to deltopectoral crest, tuber on anterolateral margin: absent (0); present (1). (Loewen et al. 2013).

787): Maxilla, antorbital fossa, ventral margin, depth: decreases anteroposteriorly (0); uniform along most of its length (1). (Modified from Brusatte et al. 2010).

788): Nasal, posterior (frontal) processes, number: no more than 2 (0); more than 2 (1). (Brusatte et al. 2010).

789): Surangular, accessory posterior foramen: absent (0); present (1).

792): Manual unguals, collateral grooves, form: simple, unforked (0); proximally forked, producing a pair of distally converging furrows (1).

793): Premaxillary teeth, cross section, major axis of elongation: mesiodistal (0); labiolingual (1).

796): Manual phalanx P1-I, proximodistal length: subequal to or less than (0); more than (1) the length of manual phalanx P1-II.

797): Dorsal vertebrae, posterior postzygapophyses, distalmost extent: at the same level or anteriorly to (0); posteriorly to (1) the posterior end of the centrum. (Maryańska et al. 2002).

798): Preorbital skull, antorbital fenestra, proportions: taller than long or as long as tall (0); longer than tall (1). (Modified from Senter et al., 2004).

800): Mt II and IV, proximal end, anterior view, contact: absent (0); present (1). (Holtz 2000).

801): Frontal, supraorbital rim: absent (0); present (1). (Senter 2010).

803): Preacetabular process, anteroposterior length: no more than (0); more than (1) 6/5 of its proximal dorsoventral height. The proximal height of the preacetabular blade is measured at the level of the anterodorsal margin of the pubic peduncle of the ilium.

804): Femur, proximal articulation, shape in anteromedial view: rounded or convex (0); flattened (1).

805): Frontal, supratemporal fossa, anterior margin, shape: straight or slightly curved (0); strongly sinusoidal (1). (Senter 2010).

806): Humerus, shaft between deltopectoral crest and distal condyles, proximodistal length: subequal to or less than (0); more than (1) 5 times minimal shaft diameter. (Modified from Senter 2010).

807): Ulna, shaft, shape in lateral/medial view: straight or slightly sigmoid (0); posteriorly bowed (1). (Gauthier 1986).

809): Astragalus, articular facet for calcaneum, anterolateral notch: absent (0); present (1).

810): Astragalus, posterolateral ascending process: absent (0); present (1). (Agnolin et al. 2010).

811): Astragalus, ascending process, lateral margin, distinct vertical ridge marking the fibula facet: absent (0); present (1).

812): Teeth, labial and lingual surfaces, texture: smooth (0); series of slight wrinkles oriented mesiodistally (1). (Modified from Benson et al. 2010).

813): Dorsal vertebrae, hyposphene, step-like ridges on lateral margin: absent (0); present (1). (Smith et al. 2008).

814): Surangular, posterior end, lateral groove: absent (0); present (1). (Smith et al. 2008).

815): Ilium, postacetabular process, brevis shelf (lateroventral crest), development: diminishes anteriorly (0); developed anteriorly (1). (Langer and Benton 2006).

817): Maxillae, lateral surfaces, orientation toward each in dorsal view: acutely angled (0); subparallel (1): (Rauhut 2003).

818): Frontal, dorsal surface, anterolateral corner of orbital margin, dorsal prominence/horn: flat, process absent (0); convex, process present (1). (Cau et al. 2013).

819): Premaxilla, maxillary process, orientation in lateral and anterior views: faces laterodorsally (0); lies flat in the horizontal plane (1). (Brusatte et al. 2010).

820): Squamosal, quadratojugal process, distal end, shape in lateral/medial view: pointed (0); blunt (1). (Brusatte et al. 2010).

821): Premaxilla, lateral surface: smooth (0); pierced by neurovascular foramen/foramina (1). (Modified from Ezcurra and Novas 2007)

823): Opening for the internal carotid artery: not bordered (0), bordered (1) by a pneumatic fossa. (Currie and Carpenter 2000; Holtz 2000)

824): Maxilla, medial surface, inflated/swollen bulla vestibularis: absent (0); present (1).

825): Mt III, shaft, proximal half, posterior margin, shape: broad to rounded (0); mediolaterally constricted/sharp (1).

829): Mc III, distal articulation: ginglymoid (0); convex (1). (Senter 2010).

831): Distal carpal 1+2 block, articulation with Mc II: articulates (0); fails to articulate (1) with the lateral half of the proximal surface of Mc II. (Modified from Senter 2010).

835): Cervical vertebrae, postaxial postzygapophyses, anteroventral surface: lacking foramina (0); pierced by foramen/foramina (1). (Tykoski 2005).

836): Fibula, distal end, medial flange overlapping the ascending process of the astragalus: absent (0); present (1). (Tykoski 2005).

838): Cervical vertebrae, posterior neural arches, hyposphene-like accessory articulation: absent (0); present (1). (Smith et al. 2008).

839): Maxillary teeth, apicobasal height: highly variable with gaps evident for replacement (0); almost isodont, with no more than a 30% difference in height between adjacent teeth, and with no replacement gaps (1). (Senter 2010).

840): Maxillary/post-symphyseal dentary crowns, lingual surface bearing apicobasally directed ridges/flutes: absent (0); present (1).

841): Premaxilla, medial surface, foramen placed below the narial margin: absent (0); present (1). (Modified from Wilson et al. 2003).

842): Quadratojugal, sharp lateral flange running anterodorsally: absent (0); present (1). (Smith et al. 2008).

843): Cranial nerve V, opening, position: not posteriorly (0); posteriorly (1) to the apex of nuchal (supraoccipital) crest. (Modified from Coria and Currie 2002).

844): Prootic, foramen of facial nerve (Cranial nerve VII), shape: round or slightly anteroposteriorly elongate (0); dorsoventrally elongate (1). (Smith et al. 2008).

845): Cranial nerve VI, median ridge separating exits: present (0); absent (1). (Coria and Currie 2002).

846): Ectopterygoid, ventral recess, shape: medial depression/groove (0); foramen leading from the medial side laterally into the body of the ectopterygoid (1). (Modified from Gauthier 1986).

847): Cervical vertebrae, pleurocoels, suboval, long axis anteroposteriorly oriented (0); slit-like, posteroventrally inclined (1). (Smith et al. 2008).

850): Mt IV, distal end, shape in distal view: as broad as deep (0); deeper than broad (1). (Modified from Sereno 1999).

851): Dorsal vertebrae, anterior neural spines, inclination: dorsally or posteriorly directed (0); anterodorsally directed (1).

852): Premaxilla, nasal process, posteriormost extent: at the same level or anteriorly (0); posteriorly to (1) the posterior tip of the ventral (maxillary) process of premaxilla. (Yates 2006).

853): Quadrate, proximodistal (dorsoventral) diameter: more than (0); subequal to or less than (1) 2 times the mediolateral diameter of its distal articulation.

855): Tibia, proximal end, lateral margin of the lateral condyle, shape in proximal view: uniformly convex (0); indented (1). (Modified from Brusatte et al. 2010).

856): Fibula, proximal (dorsal) margin, shape in medial/lateral view: straight or slightly convex (0); concave, anteriorly upturned (1). (Brusatte et al. 2010).

857): Tibia, proximal end, lateral condyle, anteroposterior extension in lateral view: short, does not reach the anterior margin of the tibial shaft (0); long, reaching the anterior margin of the tibial shaft (1). (Brusatte et al. 2010).

859): Maxilla, region ventral to external naris, dorsal margin, inclination in lateral view: inclined and facing anterodorsally (0); subhorizontal and facing dorsally (1).

861): Dorsal vertebrae, prezygoparapophyseal lamina: absent (0); present (1). (Yates 2006).

862): Dorsal vertebrae, middle parapophyses, position: ventrally to (0); at the same level to (1) diapophyses. (Yates 2006).

863): Postorbital, posteroventral margin, shape in lateral/medial view: sharply flexed (0); gently concave (1). (Novas et al. 2008).

864): Maxilla, form of articular surface for nasal anteroventral process, and form of nasal anteroventral process: tapered process (0); blunt-tipped anteroventral process (1). (Modified from Sereno and Brusatte 2008).

865): Maxilla, posterior process, inclination of ventral margin under jugal articulation (lateral view): horizontal (0); declined by approximately 20° (1). (Sereno and Brusatte 2008).

866): Maxilla, ventral margin, position of lateral rim relative to the medial rim: ventral (0); at the same level or dorsal (1). (Modified from Holtz et al. 2004).

868): Caudal vertebrae, neural canal, mediolateral diameter: no more (0); more (1) than 1/3 of centrum proximal height.

870): Tibia, distal end, articular facet for ascending process of astragalus subdivided by a proximodistally elongate process, and corresponding sulcus on the posterior surface of the ascending process of astragalus: absent (0); present (1).

871): Maxilla, ventral margin of the antorbital fossa: narrower than the ventral process of the maxilla (0); deeper than the ventral process of the maxilla (1).

872): Lacrimal, posterodorsal process, cornual boss: absent (0); present (1). (Loewen et al. 2013).

873): Maxilla, dorsal process, anterodorsal margin, shape in lateral view: arched, dorsally convex (0); angular (1).

874): Nasals, relationships: unfused (0); fused (1).

875): Prefrontal: present (0); absent (1).

876): Jugal, quadratojugal process, posterodorsal process, anteroposterior length: less than (0); subequal to or more than (1) the posteroventral process.

877): Interorbital septum, extensive ossification: absent (0); present (1). (Coria and Currie 2002).

878): Quadrate, quadratojugal contact in posterior view, cleft of the quadrate foramen, dorsoventral axis: less than (0); more than (1) 1/3 ventral width of quadrate.

879): Quadrate, quadratojugal contact in posterior view: interrupted by a cleft (0); continuous (1).

880): Quadratojugal and quadrate: unfused (0); fused (1).

882): Palatine, jugal process, distal expansion: absent (0); present (1). (Currie and Carpenter 2000).

884): Maxillary/dentary teeth, serration, distal density / mesial density index: less than (0); more than (1) 1.25.

885): Dentary teeth, first tooth, size: comparable to (0); smaller than (1) teeth 4^th^ to 6^th^.

887): External mandibular fenestra: present (0); absent (1).

888): Dentary, posterior half, medial paradental sulcus separating the interdental septa from the lingual bar: absent (0); present (1).

889): Dentary, medial surface, foramina at the anterior end of the Meckelian groove, number: one (0); two (1). (Benson et al. 2010).

891): Sacral vertebrae, centra, ventral surface, longitudinal sulcus: absent (0); present (1).

892): Splenial, mylohyoid foramen, size: small (0); wide fenestra (1).

893): Splenial, mylohyoid foramen, shape: anteroventrally opened notch (0); closed foramen (1).

895): Dorsal vertebrae, posteriormost centra, parapophyses, position: ventrally to (0); at the same level and joined to (1) the prezygodiapophyseal lamina. (Coria and Salgado 2000).

896): Scapula, distal end, distinct “shoulder” (dorsoventral expansion) relative to rest of shaft: present (0); absent (1).

899): Ulnare: present (0); absent (1) distally to the ulna.

905): Postacetabular process, posterior margin, posterodorsal process projected posteriorly beyond level of mid-height of posterior margin: absent (0); present (1).

907): Ischium, mediodorsal process, shape: simple tubercle (0); elongate crest (1).

908): Femur, shaft, surface area placed distally to the anterior trochanter: smooth (0); bearing a proximodistally elongate ridge (1).

909): Tibia, fibular crest, relationship with the proximal condyles of the tibia: separated (0); joined (1). (Modified from Rauhut 2003).

911): Astragalus, fibular articular facet, orientation: proximolaterally (0); laterally (1). (Modified from Rauhut and Xu 2005).

913): Paroccipital process, dorsal margin, shape: straight (0); twisted anterolaterally at distal end (1). (Senter 2010).

914): Maxilla, maxillary recess, medial wall: unfenestrated (0); fenestrate and leading to maxillary antrum (1).

915): Nasal, posterior end, shape in dorsal view: the medial projections extend as far or further posteriorly than the lateral projections (0); the lateral projections extend further posteriorly than the medial projections (1). (Holtz et al. 2004).

916): Dentary, anterodorsal margin, shape in lateral/medial view: angled (0); strongly beveled (1). (Modified from Senter 2010).

917): Caudal vertebrae, median neural spines, inclination of dorsoventral axis: posterodorsally (0); subvertical (1).

919): Parietal, nuchal plate, orientation with respect to frontal–parietal–postorbital suture: not parallel (0); parallel (1). (Modified from Coria and Currie 2002).

920): Supraoccipital, participation to the dorsal margin of the foramen magnum: present (0); absent (1).

922): Mc I, proximolateral margin, shape in dorsal/ventral view: continuous with the proximomedial face (0); strongly sloped laterally (1).

923): Astragalus, ascending process, angle between the proximomedial corner and the transverse axis of the astragalus: no more than (0); more than (1) 45°.

924): Radius/ulna (excluding olecranon process), proximodistal diameter: subequal to or more than (0); less than (1) 6 times its mid-shaft diameter.

929): Caudal vertebrae, anterior and median ribs, major axis of elongation, length: less (0); more (1); than 7/5 of the length of the centrum. (Modified from Canale et al. 2008).

930): Caudal vertebrae, anterior and median neural arches, space between the prezygapophyses and the base of the neural spine, shape: narrow prespinal fossa (0); narrow and robust prespinal lamina bordered laterally by the spinozygapophyseal laminae (1).

931): Humerus, anterodistal end, distinct brachial fossa: absent (0); present (1). (Modified from O’Connor 2009).

942): Dorsal vertebrae, anterior neural arches, base of prezygapophyses, pneumatic recesses: absent (0); present (1).

943): Dorsal vertebrae, prespinal and postspinal laminae, dorsal extent: terminate at the same level to (0); ventrally to (1) of neural spine. (Modified from Senter 2010).

945): Mc III, proximal end, position relative to the proximal end of Mc II: laterally (0); ventral (1). (Modified from Senter 2010).

946): Premaxillary teeth, pattern of arrangement: aligned, not overlapping (0); partially overlapping en-echelon (1).

947): Maxilla, paradental plates, exposition in medial view: relatively tall, broadly exposed (0); low and partially obscured by lamina of maxilla (1). (Modified from Carrano and Sampson 2008).

948): Radius and ulna, distal ends, shape: mediolaterally unexpanded and flattened (0); mediolaterally expanded and hemispherical (1). (Tykoski 2005).

949): Postorbital, ventral (jugal) process, posterior margin, inclination relative to dorsal surface on lateral view: perpendicular (0); anteroventrally directed, forming an angle of more than 25° (1). (Pol and Rauhut 2012).

950): Ilium, postacetabular process, notch between the supracetabular crest and the ventrolateral margin of the postacetabular blade: present (0); absent (1). (Modified from Tykoski 2005).

951): Maxilla, articular surface with the premaxilla, inclination in lateral view: angled strongly posterodorsally (0); subvertical (1). (Brusatte and Sereno 2008).

952): Maxilla, anterior paradental plates, dorsoventral depth: less (0); or more (1) than 3/2 their antero-posterior width. (Modified from Brusatte and Sereno 2008).

953): Squamosal, ventral (= precotyloid) process, length relative to the posterior (= postcotyloid) process in lateral view: longer (0); subequal (1). (Brusatte and Sereno 2008).

954): Dentary, posterior end of principal neurovascular foramina row, location: parallels the tooth row (0); curves ventrally as it extends posteriorly (1). (Brusatte and Sereno 2008).

955): Gastralia, distal end of medial element, shape: tapered (0); club-shaped prominence (1). (Brusatte and Sereno 2008).

956): Gastralia, number of sets of fused medial elements: zero or one (0); more than one (1). (Brusatte and Sereno 2008).

957): Ilium, anterior margin of preacetabular process, profile: gently convex (0); subvertical, straight (1). (Brusatte and Sereno 2008).

958): Ischium, posteriorly-directed flange on iliac peduncle: absent (0); present (1). (Brusatte and Sereno 2008).

959): Femur, lateral distal condyle, form: flat to bulbous (0); cone-shaped (1). (Brusatte and Sereno 2008).

960): Nasal, premaxillary process, anterodorsal end, notch: absent (0); present (1). (Brusatte et al. 2010).

961): Nasal, transverse section, shape: uniformly convex (0); “D”-shaped (1). (Brusatte et al. 2010).

962): Nasal, posterolateral process, exposition: present (0); covered by the lacrimal (1). (Carr 2005).

963): Maxillary fenestra, anteroposteror axis: less than twice height (0); more than twice (1) height.

964): Nasal, posteromedial process: present (0); absent (1). (Brusatte et al. 2010).

965): Femur, tibiofibular crest (ectocondylar tuber), shape and orientation in posterior view: narrow, longitudinal (0), broad, oblique (1). (Carrano and Sampson 2008).

966): Skull, postorbital, lacrimal and jugal, lateral surfaces: smooth (0); sculptured (1). (Carrano and Sampson 2008).

968): Nasal–frontal contact, posteriormost extent, position: anterior to base of nasal process (0); at the level of or posterior to base of nasal process (1). (Modified from Carrano and Sampson 2008).

969): Supraoccipital, couple of foramina for middle cerebral vein on either side of posterior supraoccipital crest, position: laterally spaced (0); closely appressed medially (1). (Tortosa et al. 2013).

971): Postorbital–squamosal contact, appearance in lateral view: contact edges visible (0), edges covered by dermal expansions (1). (Carrano and Sampson 2008).

972): Lacrimal, antorbital fossa, exposition: exposed laterally (0), covered by dermal ossifications (1). (Carrano and Sampson 2008).

973): X cranial nerve opening, position: through otoccipital (0); onto occiput (1). (Carrano and Sampson 2008).

975): Splenial, anterior end, prongs, number: one (0), two (1). (Carrano and Sampson 2008).

976): Dentary, lateral groove, position: at mid-height or dorsally (0), in ventral half (1). (Carrano and Sampson 2008).

977): Cervical vertebrae, transverse processes, dorsal surface, accessory fossa: present (0), absent (1). (Carrano and Sampson 2008).

978): Dorsal vertebrae, paradiapophyseal lamina, development: poorly developed (0); pronounced (1). (Carrano and Sampson 2008).

979): Femur, laterodistal end, laterally protruding prominence: absent (0); present (1).

980): Cervical ribs, shaft bifurcation: absent (0), present (1). (Carrano and Sampson 2008).

981): Coracoid, posteroventral process, proximodistal diameter: less than (0); more than (1) twice the diameter of the glenoid. (Modified from Carrano and Sampson 2008).

982): Pubis, foot, dorsal surface, mid-line shape: convex (0); concave (1). (Carrano and Sampson 2008).

983): Fibula, insertion of M. iliofibularis, size: moderate (0), large (1). (Carrano and Sampson 2008).

984): Lacrimal, lateral dorsal recess, anteroposterior diameter in lateral view: no more than (0); at least (1) two times its posterior height. (Brusatte et al. 2010).

985): Lacrimal, lateral dorsal recess, dorsoventral diameter: no more than (0); more than (1) the dorso-ventral diameter of the lacrimal above the recess. (Brusatte et al. 2010).

986): Lacrimal, medial recess: absent (0); present (1). (Brusatte et al. 2010).

987): Postorbital, orbital margin of the adult, shape in lateral view: straight (0); concave (1). (Modified from Brusatte et al. 2010).

988): Preorbital skull, antorbital fenestra length: less than (0); subequal to or more than (1) 1/4 skull length. (Tykoski 2005).

990): Caudal vertebrae, anterior and median chevrons, proximoposterior process, development: indistinct (0); pronounced (1). (Modified from Sereno 1999).

993): Angular, exposition in lateral view: exposed almost to end of mandible, reaches or almost reaches articular (0); excluded from posterior end of articular, suture turns ventrally and meets ventral border of mandible anterior to glenoid (1). (Senter 2010).

994): Premaxillo-maxilla suture, lateral view, fenestra at the level of the external naris (dorsal to subnarial foramen, when present): present (0); absent (1).

997): Mt V, shape: straight (0); anterodistally curved (1). (Modified from Rauhut 2003).

998): Fibula, proximal end, width: less than 3/4 (0); more than 3/4 (1) of the proximal width of the tibia. (Holtz 2000).

999): Tibia, distal end, medial malleolus, development: mediolateral development: less than (0); more than (1) its proximodistal extent. (Modified from Sereno 1999).

1000): Femur, distal end, mediodistal process, development: ridge (0); broad flange/shelf (1). (Modified from Carrano and Sampson 2008).

1002): Lacrimal, ventral process, lateral foramen, position: near the base (0); at mid-height (1). (Sereno 1999).

1003): Humerus, proximal end, posterior surface, capital incisure between head and internal (medial) tuberosity: absent (0); present (1).

1008): Anterior presacral centra, anterior pneumatic recess, number of openings: single opening (0); multiple openings (1). (Modified from Harris 1998; Brusatte and Sereno 2008).

1010): Ulna, lateral tuberosity, development: small mound (0); hypertrophied and robust (1). (Smith et al. 2008).

1011): Manual phalanx P1-I, ventral surface, shape: relatively flat or weakly concave (0); strongly concave with deep ventral furrow (1). (Smith et al. 2008).

1012): Radius, proximoanterior process, development: reduced (0); prominent, subtriangular in proximal view (1). (Smith et al. 2008).

1013): Radius, posteromedial edge, ulnar process at mid-length: absent (0); present (1).

1014): Basioccipital, ventral recess: absent (0); present (1). (Modified from Senter 2010).

1016): Caudal vertebrae, pre- and postspinal laminae: absent (0); present (1).

1019): Mc III, diaphysis, length: more (0); subequal or less (1) than 2 times distal epiphysis width.

1020): Maxillary fenestra, shape: rounded (0); crescentic/slit-like (1).

1021): Tibiotarsus, distal end, posterior extension of articular surface for distal tarsals/tarsometatarsus: absent, articular restricted to distalmost edge of posterior surface (0); well-developed posterior extension, *sulcus cartilaginis tibialis* (*sensu* Baumel and Witmer 1993), distinct surface extending up the posterior surface of the tibiotarsus (1). (Clarke and Norell 2002).

1024): Ilium, preacetabular process, medial ridge, development: slightly reduced (0); prominent (1). (Holtz et al. 2004).

1025): Humerus, head, long axis in proximal view: collinear with the plane of the proximal expansion of the humerus (0); oriented slightly obliquely (1).

1026): Mc I, proximal end, lateral proximolateral process: poorly developed (0); prominent (1).

1029): Postorbital, medial surface, articular facet for the laterosphenoid, shape and development: shallow (0); deep concavity (1). (modified from Sereno and Brusatte 2008).

1032): Caudal vertebrae, median ribs, shape in dorsal/ventral: narrow-based and subrectangular (0); wide-based and prominent, alariform (1). (Novas et al. 2004).

1033): Caudal vertebrae, median and posterior ribs, dorsal surface: flat (0); excavated (1). (Novas et al. 2004).

1035): Caudal vertebrae, anterior neural spines, shape: sheet-like (0); rod-like (1). (Carrano and Sampson 2008).

1036): Fibula, proximomedial end, fossa/groove, posterior margin: closed by a lip (0); open (1). (Modified from Carrano and Sampson 2008).

1037): Sternum, plates in articulated adult specimens: unossified (0); ossified (1).

1041): Frontal, ventral surface, olfactory bulbs, position: widely spaced (0); closely appressed medially (1).

1043): Orbitosphenoid: present/ossified (0); absent/unossified (1). (Holtz et al. 2004).

1044): Frontal, interfrontal suture in adults: open, visible (0); closed, coossified (1). (Holtz 2000).

1049): Skull, elongation in adult: shorter (0); longer (1) than 3 times the occipital height. (Sereno 1999).

1050): Premaxillary-maxillary oral margin: continuous (0); interrupted by a gap (1).

1052): Cervical vertebrae, centra 7–9, proximodistal length: subequal to (0); more than (1) 10% of the length of the axis. (Yates 2003; modified from Gauthier 1986).

1053): Frontal-postorbital facet, anterior depth: less (0); more (1) than 2/5 facet length.

1055): Ilium, preacetabular process, anteroventral corner with distinct ventral projection (antiliac process): absent, anteroventral margin rounded (0); present, anteroventral margin acuminate (1).

1056): Ilium, pubic peduncle, posterodistal margin of lateral surface, mound-like eminence: absent (0); present (1).

1057): Olfactory bulbs, greatest diameter: length (0); depth (1). (Zelenitsky et al. 2008).

1058): Cerebral hemisphere, greatest diameter: depth (0); length (1). (Zelenitsky et al. 2008).

1059): Olfactory ratio (%): more than 45 (0); less than 45 (1). (Zelenitsky et al. 2008).

1061): Dorsal vertebrae, anterior neural arches, hyposphene-hypantrum articulation: absent (0); present (1).

1062): Manual ungual I, flexor tubercle, ventral surface, transverse groove: absent (0); present (1).

1063): Femur, tibiofibular crest (ectocondylar tuber), posteriormost extent in distal view: anteriorly to the posteriormost extent (0); at the same level or more posteriorly than (1) the posteriormost extent of the medial condyle.

1064): Astragalus, parapet anterior to ascending process and ascending process base, depression: absent (0); present as a semilunate fossa (1). (Rauhut and Xu 2005).

1065): Pedal unguals, ventral fossa: absent (0); present (1).

1066): Pedal unguals II and IV, marked asymmetry among the external surfaces: absent (0); present (1).

1067): Pedal digit IV, intermediate phalanges, shape: longer than broad (0); broader than long (1).

1069): Pedal unguals, collateral groove, confluence with the ventral surface: absent (0); present (1).

1070): Mt I, shape: long and slender, longer than 4 times its distal width (0); short and robust, long no more than 4 times its distal width (1).

1071): Mc II and III, mediodistal condyle, marked laterodistal lips: poorly developed (0); present, directed proximomedially (1).

1072): Cervical vertebrae, middle centra, posterior surface, mediolateral width: less than (0); subequal to or more than (1) 6/5 of the dorsoventral diameter of the same surface. (Modified from Sereno 1999).

1073): Dorsal vertebrae, neural spines, spinodiapophyseal basal webbing: absent (0); present (1). (Modified from Sereno 1999).

1074): Dorsal vertebrae, anterior neural spines, shape: longer than tall or as tall as long (0); taller than long (1).

1075): Basioccipital, posterior surface, median vertical crest: present (0); absent (1). (Canale et al. 2008).

1076): Premaxilla and maxilla, paradental laminae, depth along the tooth row: increases anteriorly since the posterior end of the toothrow (0); homogeneous along all the tooth row length (1). (Canale et al. 2008).

1077): Maxilla/jugal articulation, inclination in lateral view: less than (0); subequal or more than (1) 45° relative to ventral margin. (Canale et al. 2008).

1078): Splenial, anteroventral process, length relative to the anterodorsal one: larger or subequal (0), less (1). (Modified from Canale et al. 2008).

1079): Jugal, ventral margin, shape in lateral/medial view: nearly flat or slightly convex (0); strongly convex (1). (Canale et al. 2008).

1080): Skull, supratemporal fenestra, proportions: longer than wide or as long as wide (0); wider than long (1). (Modified from Canale et al. 2008).

1081): Postorbital, dorsal margin, inflation: absent (0); strongly developed (1). (Canale et al. 2008).

1083): Cervical vertebrae, postzygapophyses, elongation: posteriorly short, not surpassing the posterior end of the vertebral centra (0); swept back posteriorly widely surpassing the posterior end of vertebral centra (1). (Carrano and Sampson 2008).

1084): Axis, pleurocoels, position: ventrally to (0); posteriorly to (1) the diapophyses. (Canale et al. 2008).

1085): Axis, postzygodiapophyseal lamina, development: poorly developed (0); prominent (1). (Canale et al. 2008).

1086): Cervical vertebrae, diapophyses, shape: rod-like and anteroposteriorly narrow (0); with anteroposteriorly extended lateral surfaces (1). (Canale et al. 2008).

1087): Dorsal vertebrae, neural arch base, shape: dorsoventrally low and laterally expanded (0); dorsoventrally tall and laterally compressed (1). (Canale et al. 2008).

1089): Manual non-ungual phalanges, distal surfaces, development: well-defined condyles (0); flattened (1). (Modified from Canale et al. 2008).

1090): Mt III, distal end, shape: ginglymoid, dorsoventrally extended, distinct from mt shaft (0); mediolaterally wide and dorsoventrally low, being its dorsal margin continuous with shaft when viewed laterally (1). (Modified from Novas et al. 2004).

1092): Maxilla, preantorbital process, lateral subcutaneous surface, proportion: taller than long (0); longer than tall (1).

1095): Lacrimal, posterodorsal process, orientation: perpendicular (0); posterodorsally inclined to subvertical (1). (Senter 2010).

1097): Retroarticular process, direction: points posteriorly (0); curves posterodorsally (1). (Senter 2005).

1105): Ulna, robustness relative to tibiotarsus: significantly more slender than (0); as robust as (1) tibiotarsus. (Xu et al. 2009).

1108): Ulna, proximal third of shaft, anterior margin, thick ridge from coronoid (anterior/sigmoid) process: absent (0); present (1). (Smith et al. 2008; Xu et al. 2008).

1109): Ulna, distal end, proximal extension of distal facet along the lateral margin: weak, distal margin nearly straight in posterior view (0); significant, distal margin convex in posterior view (1). (Modified from Xu et al. 2009).

1113): Tibia, lateral cnemial crest: poorly developed (0); prominent (1).

1115): Surangular, anterior foramen in groove, development: smaller than groove (0); as large as groove (1).

1116): Pedal unguals, dorsal surface, shape: continuously convex (0); with dorsoproximal concavity (1). (Brusatte et al. 2010).

1117): Dorsal vertebrae, posterior centra, ventral keel: absent (0); present (1).

1118): Distal carpals 1-2 in adult articulated specimens: present, ossified (0); absent, unossified (1).

1123): Dentary, anteroventral margin, form: smooth, convex (0); marked by a projecting flange, forming a ‘dentary chin’ (1). (Brusatte and Sereno 2008).

1124): Odontoid, foramen/depression on anterolateral surface: absent (0); present (1). (Brusatte et al. 2008).

1125): Axis, neural spine, lateral foramen/foramina: absent (0); present (1). (Brusatte et al. 2008).

1126): Post-axial cervical centra, marked rim around the anterior convexity in opisthocoelous forms: absent (0); present (1).

1127): Dorsal vertebrae, hyposphene, shape in posterior view: subtriangular (0); rectangular (1). (Modified from Brusatte et al. 2008).

1128): Post-axial cervical vertebrae, epipophyses, position: distally on postzygapophyses, dorsal to postzygapophyseal facets (0); placed proximally, anterior to postzygapophyseal facets (1). (Senter 2010).

1129): Sacral vertebrae, neural spine, pneumaticity: absent (0); present (1). (Carrano and Sampson 2008).

1130): Cervical vertebrae, hypapophyses in posterior centra, development: poorly developed (0); prominent (1).

1131): Postorbital, anterodorsal process, slenderness: not elongate and robust (0); very elongate and slender (1).

1132): Femur, anterior cleft between anterior and greater trochanters, elongation: short, less than half (0); elongate, more than half (1) dorsoventral depth of femur head.

1134): Furcula, symphysis, cross section: rounded (0); anteroposteriorly compressed (1). (Nesbitt et al. 2009).

1135): Ischium, distal expansion, anteroposterior diameter: less (0); more (1) than twice minimum ischial shaft anteroposterior diameter.

1139): Scapula, acromion, anteroposterior extent: less (0); more (1) than twice proximal shaft depth.

1143): Ilium, pubic peduncle, dorsoventral depth to basal anteroposterior length ratio: deeper than long (0); longer than deep (1). (Modified from Longrich and Currie, 2009).

1146): Premaxilla-maxilla articulation, shape in lateral view: simple (0); interdigitate (1). (Benson et al. 2010).

1149): Tibia, distal end, medial malleolus, shape in anterior/posterior view: angular or rounded (0); truncated (1).

1150): Postorbital bar, anteroposterior diameter at mid-height: subequal to (0); more than (1) the anteroposterior diameter of the lacrimal at mid-height. (Brusatte et al. 2010).

1151): Basisphenoid, pronounced muscle scar flanking the ventral recess: absent (0); present (1). (Brusatte et al. 2010).

1152): Quadratojugal, posterior border, posteroventral overlapping the quadrate: absent (0); present (1). (Modified from Brusatte et al. 2010).

1153): Mt III, distal half, dorsal view, shape of medial margin: straight (0); bearing a medial expansion/bulge (1).

1154): Occipital condyle, distinct neck: absent (0); present (1).

1155): Maxilla, orientation of the groove for the dental lamina (paradental groove) on the medial surface: horizontal across its length (0); horizontal for most of its length but curves ventrally at its anterior extent (1). (Benson et al. 2010).

1156): Braincase, facial (VII) nerve foramen, number: one (0); two (1). (Benson et al. 2010).

1157): Braincase, fenestra ovalis, primary orientation: medio-lateral, such that it opens on the lateral wall of the braincase (0); antero-posterior, and located on the web of bone linking the crista tuberalis and the paroccipital process, such that it opens mostly anteriorly (1). (Modified from Coria and Currie 2002).

1158): Axis, centrum, anteroposterior length: elongate, longer than 1.2 times the height of the posterior articular face (0); short, less than 1.1 times the height of the anterior articular face (1). (Benson et al. 2010).

1159): Ilium, postacetabular process, medioventral shelf, development: developed as a ridge (0); prominent shelf ventrally projected (1).

1160): Astragalus, lateral condyle, antero-proximal extension of the articular face as a rounded triangular process: absent (0); present (1).

1161): Ischium, obturator notch (in taxa with a ventrally opened notch), shape in lateral/medial view: “U”-shaped (0); with diverging sides (1). (Modified from Holtz et al. 2004).

1162): Cervical vertebrae, interpostzygapophyseal lamina: absent (0); present at least in anterior vertebrae (1). (Zanno 2010).

1163): Mc I, mediodistal condyle, development: well formed (0); rudimentary (1). (Brusatte et al. 2010).

1164): Humerus, distal end, medial condyle, mediolateral width: comparable (0); larger (1) than lateral condyle. (Brusatte et al. 2010). (Char. # 1164).

1165): Dorsal vertebrae, neural spines, dorsal view: quadrangular/trapezoid (0); spine anteriorly and posteriorly bifurcated, medially pinched in dorsal view (1). (Zanno 2010).

1167): Humerus, distal half, anterolateral margin, groove ascending dorsal to medial epicondyle: absent (0); present (1). (Zanno 2010).

1168): Ilium, pubic peduncle, shape of cross section: quadrangular (0); roughly triangular in outline (1). (Modified from Zanno 2010).

1170): Ischium, articular surface for ilium, shape: flat or slightly concave (0); iliac peduncle of ischium with deep cavity for insertion of peg-shaped, ventrally tapering ischiadic peduncle of ilium (1). (Carrano and Sampson 2008; Zanno 2010).

1171): Fibula, proximal end, anterior and posterior margins: subequal in transverse width (0); transverse width of proximal fibula narrows posteriorly (1). (Zanno 2010).

1172): Dorsal vertebrae, middle and posterior postzygapophyses, small, flange-like lateral extensions of postzygapophyseal facets: absent (0); present (1). (Benson et al. 2010).

1173): Coracoid, lateral fossa ventrodistal to glenoid (subglenoid fossa): absent (0); present (1). (Benson et al. 2010).

1174): Ilium, large external pneumatic foramina and internal spaces: absent (0); present (1). (Benson et al. 2010).

1175): Maxilla, promaxillary recess, exposition in lateral view: present (0); absent (1).

1176): Tibia, proximolateral condyle, anterolateral process, orientation: horizontally projected (0); curves ventrally (1). (Modified from Benson et al. 2010).

1177): Ulna, posterior surface distal to olecranon process: rounded (0); sharp (1). (Smith et al. 2008).

1178): Atlas, neural arch, pneumatic foramen in dorsolateral surface: absent (0); present (1). (Benson et al. 2010).

1179): Femur, distal end, morphology: central depression connected to crista tibiofibularis by a narrow groove (0); anteroposteriorly oriented shallow trough separating medial and lateral convexities (1). (Benson et al. 2010).

1180): Astragalus, fibular articular facet, orientation: proximolaterally (0); laterally (1). (Modified from Rauhut and Xu 2005).

1181): Squamosal, dorsotemporal fossa: absent or flat (0); convex (1). (Brusatte et al. 2010).

1182): Squamosal, pneumatic sinus: absent (0); present (1). (Brusatte et al. 2010).

1183): Ectopterygoid, jugal process: not inflated (0); inflated (1). (Brusatte et al. 2010).

1184): Ectopterygoid, surface adjacent to pneumatic recess: flat (0); lip (1). (Modified from Brusatte et al. 2010).

1185): Caudal vertebrae, anterior neural arches, pneumatic recesses: absent (0); present (1).

1186): Ilium, antiliac (anteroventral) process, shape in lateral view: subtriangular (0); half-crescentic, with a distinct posterior concavity (1).

1187): Maxilla, pneumatic region on medial side posteroventral to maxillary fenestra: absent (0); present (1). (Benson et al. 2010).

1188): Maxilla/dentary, paradental laminae: extend apically as far as (0); fall more basally than (1) ventral level of lateral wall of maxilla. (Benson et al. 2010).

1189): Nasal, antorbital fossa: visible in lateral view (0); occluded in lateral view by a ventrolaterally overhanging lamina (1). (Benson et al. 2010).

1190): Quadrate, depression and foramen on medial surface, adjacent to mandibular condyle, at base of pterygoid process: absent (0); present (1). (Benson et al. 2010).

1191): Basioccipital apron, fossa ventral to occipital condyle: narrow and groove-like (0); broad depression approximately two-thirds the width of the occipital condyle (1). (Benson et al. 2010).

1192): Basipterygoid processes, position: located anterior or anteroventral to basal tubera (0); located ventral to basal tubera (1). (Benson et al. 2010).

1193): Maxillary/dentary, mesial carina, basal half, serration: present (0); absent (1). (Modified from Benson et al. 2010).

1194): Middle cervical vertebrae, pleurocoel penetrates centrum through parapophysis: no (0); yes (1). (Benson et al. 2010).

1195): Axis, parapophyses, development: poorly developed (0); prominent (1). (Benson et al. 2010).

1196): Sacrum, fenestrae between sacral neural spines: absent (0); present (1). (Benson et al. 2010).

1197): Ilium, acetabular margin of pubic peduncle: mediolaterally convex or flat (0); mediolaterally concave (1). (Benson et al. 2010).

1198): Femur, long axis of medial condyle in distal view: oriented anteroposteriorly (0); inclined posteromedially (1). (Benson et al. 2010).

1199): Tibia, proximal end, medial condyle, shape: bulbous eminence, not continuous with posterior surface of head (0); extends distally as a ridge that merges with posterior surface of proximal end (1). (Benson et al. 2010).

1200): Tibia, fibular flange shape: transversely narrow flange (0); oval mound (1). (Benson et al. 2010).

1201): Fibula, proximal end, lateral surface, posterior sulcus/trough: absent, surface convex (0); present (1). (Modified from Benson et al. 2010).

1202): Femur, distal end, lateral condyle, distalmost extent: does not project further distally than (0); projects distinctly further than (1) medial condyle. (Modified from Benson et al. 2010).

1203): Femur, distal end, muscle scar situated medially on anterior surface, development: suboval rugose patch not extending to distal end of femur (0); large oval depression, bound medially by a lamella (1). (Modified from Rauhut 2003).

1204): Manual phalanges, proximal end, ventral process, development: poorly developed (0); prominent and mediolaterally expanded (1).

1205): Manual ungual I: present (0); absent (1).

1206): Ilium, supracetabular crest, extent along the pubic peduncle: extensive (0); almost entirely excluded by the acetabular rim of the pubic peduncle (1).

1207): Cervical vertebrae, anterior and middle parapophyses, relationship with the diapophyses articular facet: well separated (0); closely placed (1).

1208): Mt II, shaft, cross section: rounded to elliptical (0); laminar, mediolaterally compressed (1).

1209): Ischium, symphysis, proximodistal extent: limited to the distal end (0); proximally expanded as an apron (1).

1210): Pramaxilla, maxilla and dentary, lateral surface, neurovascular foramina, position: well distant to the occlusal margin (0); very close to the occlusal margin (1). (Modified from Sereno and Brusatte 2008).

1211): Cervical vertebrae, diapophyses, posterior border, angle with the anteroposterior axis of the neural arch in dorsal view: less than (0); subequal to (1) 90°.

1212): Cervical vertebrae, prezygapophyses, shape of infraprezygapophyseal space in dorsal view: “V”-shaped, diverging sides (0); “U”-shaped, sub-parallel sides (1).

1214): Caudal vertebrae, anterior and median neural arch base relative to centrum proportions: smaller (0), equal or more (1). (Modified from Carrano and Sampson 2008).

1215): Parietal, participation to supratemporal fossa: present (0); absent (1). (Brusatte et al. 2010).

1221): Exoccipital, lateral vertical crest (metotic strut) placed anteriorly to both foramina of cranial nerve XII: absent (0); present (1). (Nebitt et al. 2010).

1223): Pubis, proximal end, lateral surface, texture: smooth (0), rugose (1). (Benson et al. 2010).

1224): Caudal vertebrae, median postzygapophyses, epipophyses: absent (0); present (1). (Carrano and Sampson 2008).

1225): Mc II, distal articulation: ginglymoid (0); convex (1).

1227): Caudal vertebrae, anterior neural arches, proportion: low and wide (0); narrow and tall (1).

1228): Anterior presacral centra, anterior half of centrum, lateral pneumatic recess: absent (0); present (1).

1229): Cervical vertebrae, middle neural arches, prezygapophyses and diapophyses, relative positions: prezygapophyses dorsally directed, placed above the diapophyses (0); prezygapophyses anteriorly directed, placed at the same level of the diapophyses (1).

1230): Mcs I-III, distal end, transverse lip bordering the proximal margin of the extensor surface: absent (0); present (1). (Modified from Ezcurra et al. 2010).

1231): Mcs I-III, distal end, collateral ligament pits, development: well developed and distinct (0); shallow, poorly developed (1). (Ezcurra et al. 2010).

1232): Femur, head, anteromedial surface, tuber: absent (0); present (1). (Modified from Nesbitt et al. 2009).

1234): Dorsal vertebrae, middle and posterior hyposphene, size in lateral view: less (0); comparable to (1) the postzygapophyses. (Novas et al. 2008).

1235): Dorsal vertebrae, anterior and middle neural arches, anterior infraprezygapophyseal fossae, development: shallow (0); deep (1).

1237): Caudal vertebrae, chevrons, proximal articular surface, distinct transverse ridge dividing surface into anterior and posterior facets: present (0); absent, low mounds may be present, one on each side, laterally (1). (Benson et al. 2010).

1244): Ilium, vertical crest above the acetabulum, inclination: dorsally directed (0); dorsoposteriorly directed (1). (Brusatte et al. 2010).

1245): Jugal, postorbital process, pronounced ridge on the lateral surface, which borders the postorbital posteriorly: absent (0); present (1). (Brusatte et al. 2010).

1260): Teeth, root, mesiodistal diameter along the apicobasal axis: uniform (0); markedly constricted close to the crown (1). (Barrett 2009).

1264): Antorbital fossa, external rim on anterior process of lacrimal: present (0); absent (1). (Wilson et al. 2003).

1265): Quadrate lateral flange, maximum width: approximately 1/2 of (0); subequal to (1), transverse width of distal condyles. (Wilson et al. 2003).

1266): Supraoccipital nuchal wedge and parietal alae, position of dorsal extremity: slightly (0); considerably (1) above frontoparietal skull table. (Wilson et al. 2003).

1267): Dentary-surangular articulation, form: narrow V-shaped notch (0); broad U-shaped socket (1). (Wilson et al. 2003).

1268): Dentary, medial articular prong for surangular (separate from dorsal prong that is exposed laterally): absent (0); present (1). (Wilson et al. 2003).

1269): Axis, intercentrum length: less than 1/3 (0); more than 1/3 (1), of axial centrum length. (Sereno 1999).

1270): Axis, spinopostzygapophyseal lamina, form: straight or gently concave (0); deeply notched (1). (Wilson et al. 2003).

1271): Cervical epipophyses, form: ridgelike or subconical (0); at least, mid cervical epipophyses anteroposteriorly extended with anterior corner (1). (Wilson et al. 2003).

1272): Cervical epipophyses, mediolateral thickness: robust (0); thin (1).

1273): Cervical vertebrae, middle (C4-6) neural spines, orientation: vertical (0); dorsoposteriorly inclined (1). (Wilson et al. 2003).

1274): Sacral neural arches, development of paramedian fossae: poorly developed (0); divided by vertical septa (1). (Wilson et al. 2003).

1275): Cervical ribs, form of lateral process for articulation with successive rib spine in mid cervicals: ridge (0); flange (1). (Wilson et al. 2003).

1276): Sacral vertebrae, posterior ribs, attachment position: ventral margin (0); angled toward dorsoposterior corner (1), of postacetabular process. (Sereno 1999).

1277): Tibia, distal half, crest placed distal to tibiofibular crest with flattened articular edge for fibular shaft: absent (0); present (1). (Wilson et al. 2003).

1278): Fibula, shaft ventral to tibiofibular crest, position relative to tibial shaft: lateral (0); anterior (1). (Wilson et al. 2003).

1280): Mc II, distal condyles, distal projection: subequal (0); leteral condyle more developed (1).

1281): Premaxilla, posterior half, teeth: present (0); absent (1).

1309): Jugal, postorbital process, inclination: posterodorsally (0), strictly dorsally (perpendicular to ventral margin of jugal) (1).

1310): Maxilla, anteromedial process, position: ventral, immediately dorsal to interdental plates (0); dorsal, immediately ventral to dorsal surface of maxillary anterior process (1). (Benson et al. 2010).

1311): Palpebral ossification: absent (0); present (1). (Currie and Carpenter 2000).

1312): Postorbital, anterodorsal process, participation to supratemporal fossa: present (0); absent (1). (Modified from Benson et al. 2010).

1313): Manual digit II, phalanges, number: 3 (0); 2 or less (1).

1314): Quadratojugal, ascending process apex, anteriormost extent relative to infratemporal fenestra: close to posterior margin of fenestra (0); close to anterior margin of fenestra (1). (Modified from Brusatte et al. 2010).

1315): Maxilla, promaxillary recess, position in antorbital fossa: anterior or anterodorsal margin (0); in the anteroventral corner (1). (Brusatte et al. 2010).

1316): Maxilla, maxillary fenestra, position in medial view: does not (0); does (1) abut the dorsal border of maxillary antrum. (Brusatte et al. 2010).

1317): Premaxilla, narial fossa, anterior margin: shallow (0); invaginated as a deep groove (1). (Brusatte et al. 2010).

1318): Premaxilla, anterodorsal margin at the level of the anterior margin of external naris, shape in lateral view: gently convex (0); distinctly inflected posterodorsally, describing a slightly obtuse or rigth corner (1). (Modified from Brusatte et al. 2010).

1320): Postorbital, ventral (jugal) process, cross section: robust, broader than long or as broad as long (0); flat and slender, longer than broad (1).

1321): Manual phalanx P1-I, distal end, mediolateral axis, clockwise torsion relative to the proximal surface, angle: less than (0); more than (1) 30°. (Martinez et al. 2011).

1322): Frontal, supratemporal fossa, anterior margin, crest bisecting the surface: absent (0); present (1). (Brusatte and Sereno 2008).

1323): Frontal, internal pneumatic sinus: absent (0); present (1).

1324): Maxilla, posterior view, lateromedial separation between interfenestral and postantral struts: wider (0); or narrower (1) than the combined width of interfenestral and postantral struts. (Eddy and Clarke 2011).

1326): Joined frontals, mediolateral width across the anteromedial margins of supratemporal fossae: more than (0); less than (1) 2/5 of the width of the paired frontals in that point.

1327): Frontal in adult, participation to dorsal orbital margin: estensive (0); extremely reduced or obliterated (1).

1329): Frontal, anterior margin of supratemporal fossa, orientation: mainly dorsally (0); posterodorsally (1).

1330): Frontal, nasal processes: present and elongate (0); strongly shortened (1).

1332): Frontal, dorsal surface: smooth (0); rugose (1).

1333): Frontal, lateral surface, dorsal margin: uniformly curved (0); markedly convex at the level of the lacrimal/prefrontal contact (1).

1334): Mandible, ventral margin, shape in lateral view: straight to moderately convex ventrally (0); markedly convex ventrally, with a distinct bend at the dentary-angular contact (1). (Senter 2011).

1335): Tibia, distal end, anteromedial margin, shape in distal view: rounded (0); marked by a proximodistally oriented ridge.

1336): Astragalus, proximal articular facet for fibula, extent: more than (0); less than (1), 3/10 of the transverse width. (Langer and Benton 2006).

1337): Anterior presacral centra, anterior half of centrum, pneumatic recess, development: not perforated medially (0); perforated medially (1). (Modified from Rauhut 2003).

1339): Femur, fourth trochanter, proximal and distal ends, inclination relative to shaft: similar slope (trochanter symmetrical) (0); distal margin more steeply inclined (trochanter asymmetrical) (1). (Langer and Benton 2006).

1340): Maxilla, dorsal process, posterolateral margin, antorbital fossa: exposed laterally (0); concealed by a thin lamina (1). (Sues et al. 2011).

1343): Frontal, medial margin of supratemporal fossae and presence of a posteriorly directed traingular plate of bone: subparallel, plate absent (0); diverging anteriorly, plate present (1). (Modified from Rauhut 2003).

1344): Ethmoidals: unossified (0); ossified (1).

1345): Posterior tympanic recess, position: on anterior surface of paroccipital process (0); extends into opisthotic posterodorsally to fenestra ovalis, confluent with this fenestra (1). (Modified from Senter 2010).

1346): Lacrimal, ventral process, lateral lamina, anteriormost point, position: at mid-height (0); in the dorsal half (1). (Smith et al. 2008).

1347): Dorsal vertebrae, posterior neural spines, mediolateral width at mid-height: more (0); less (1) than 1/12 of spine height.

1348): Axis, neural arch, lateral surface, pneumatic recess: absent (0); present (1).

1349): Dorsal vertebrae, anterior neural arches, lateral surface, pneumatic recesses: absent (0); present (1).

1350): Dorsal vertebrae, middle and posterior centra, pneumatic recesses: absent (0); present (1).

1351): Dorsal vertebrae, middle and posterior neural arches, lateral surface, pneumatic recesses: absent (0); present (1).

1352): Sacral vertebrae, neural arches, lateral surface, pneumatic recesses: absent (0); present (1).

1356): Dentary, interdental septa: present, at least labially (0); absent (1).

1357): Calcaneum, articulation with astragalus: open (0); fused (1).

1364): Maxilla, promaxillary recess, maximum diameter: less than (0); more than (1) 1/3 of anterior end of antorbital fossa dorsoventral depth. (Modified from Longrich and Currie 2009a).

1369): Dorsal ribs, distal end: unexpanded (0); expanded (1) relative to remaining of rib.

1370): Frontal, postorbital process, dorsal eminence: absent (0); present (1). (Modified from Carrano and Sampson 2008).

1372): Surangular, posterolateral foramen: present (0); absent (1).

1381): Accessory tympanic recess extension of the posterior tympanic recess: absent (0); present (1).

1383): Ilium, postacetabular process, lateral surface, M. ileofemoralis fossa, posteriormost extent: reachs the posterior margin of the ilium (0); stops anteriorly to the posterior margin of the ilium. (Tykoski 2005).

1385): Furcula, hypocleidum: absent (0); present (1).

1389): Maxilla, ventral process, posterior to the ascending process, ventral margin, shape: rounded (0); sharp (1). (Choiniere et al. 2010).

1390): Basipterygoid processes, lateral surface, pneumatic depression: absent (0); present (1).

1392): Mandible, internal mandibular fenestra, size: slit-like (0); wide (1).

1394): Ilium, antitrochanter: absent (0); present (1).

1396): Jugal, participation to antorbital fenestra: less than (0); subequal or longer than (1) maxilla participation.

1397): Manual non-ungual phalanges, diaphysis, length: more (0); subequal or less (1) than distal epiphysis length.

1399): Quadratojugal, ascending process, lateral surface, anterior margin, dorsoventrally oriented crest: absent (0); present (1). (Brusatte et al. 2010).

1400): Quadratojugal, jugal process, anterior end, shape in lateral view: tapered to blunt (0); forked (1). (Modified from Brusatte et al. 2010).

1401): Quadrate, distal end, lateral condyle, extension on lateral surface of bone: limited (0); extended dorsally (1). (Brusatte et al. 2010).

1402): Prefrontal-nasal contact: present (0); absent (1). (Brusatte et al. 2010).

1404): Premaxilla/maxilla, ventral view: deep sulcus between palatal shelf and paradental laminae: absent (0); present (1).

1405): Maxilla, medial view, shape of ridge across interdental plates: straight (0); sinuous (1). (Eddy and Clarke 2011).

1406): Nasal, narial fossa, posterior margin: distinct (0); covered by rugosity (1). (Modified from Eddy and Clarke 2011).

1407): Postorbital, frontal (anterodorsal) process, dorsal surface, vascular groove: absent (0); present (1). (Eddy 2008).

1408): Prefrontal-frontal facet, shape: triangular (0); rounded (1). (Eddy and Clarke 2011).

1410): Humerus, anterior surface, distal end, area at the level of lateral condyle, distinct fossa: absent (0); present (1). (Benson et al. 2010).

1411): Caudal vertebrae, anterior centrocostal lamina, anterior end: unexpanded (0); expanded anterolaterally forming a lateral spur (1). (Rauhut 2011).

1412): Caudal vertebrae, centroprezygapophyseal laminae, development: weakly developed (0); robust and leading to the neural canal (1). (Rauhut 2011).

1413): Caudal vertebrae, anterior centra, ventral surface, longitudinal sulcus, depth: shallow (0); deep with distinct margins (1). (Modified from Rauhut 2003).

1420): Maxilla, maxillary fenestra, anterior half, overlapped by lateral lamina of antorbital fossa: absent (0); present (1). (Brusatte et al. 2010).

1421): Maxilla, ventral process, lateral surface, lateral foramina, relationships: distinct (0); set in a longitudinal groove (1). (Brusatte et al. 2010).

1422): Articular, mediolateral width of jaw muscle attachment site: less than (0) or equal to more than (1) width of glenoid for articulation with quadrate (Brusatte et al. 2010).

1423): Premaxillary teeth, lingual surface: concave (0); straight (1). (Brusatte et al. 2010).

1424): Cervical vertebrae, anterior and middle centra, posterior centrodiapophyseal lamina, orientation: posteroventrally (0); posteriorly (1). (Brusatte et al. 2010).

1425): Ilium, lateral surface, dorsoventrally directed crest above the acetabulum, dorsal extent: does not (0); does (1) reach the dorsal margin of ilium.

1427): Mt IV, distal end, condyles: symmetrical (0); asymmetrical, with medial condyle prominent and lateral condyle reduced (1).

1432): Mt III, shaft, lateral surface, distal half, contact with Mt IV: absent (0); present (1).

1433): Pedal unguals lateral surface, laterally projecting ridge: absent (0); present (1). (O’Connor 2009).

1434): Maxilla, promaxillary fenestra, medial surface: unfenestrated (0); fenestrated and leading to medial surface (1).

1436): Maxilla, ascending ramus, base, lateral surface, development: large, plate-like (0); reduce lip of bone between nasal facet and antorbital fossa (1). (Modified from Turner et al. 2007).

1437): Ilium, lateral surface, vertical crest dorsal to the acetabulum, anteroposterior width: less (0); more (1) than ¼ blade height. (Modified from Brusatte and Benson 2013).

1438): Posterior dorsal and anterior caudal vertebrae, postzygapophyses, position relative to the level of the prezygapophyses: about at the same level (0); clearly dorsally (1). (Modified from Brusatte and Benson 2013).

1439): Fibula, distal end, anteroposterior length: more (0); less (1) than 9/5 mid-shaft width. (Modified from Benson et al. 2010).

1440): Pubis, foot, maximum longitudinal axis, angle formed with the distal half of pubis in lateral view: more (0); less (1) than 75°.

1441): Quadrate, shaft, proximal half, inclination relative to rest of shaft: straight (0); posteriorly curved (1). (Ezcurra and Novas 2007).

1442): Femur, anterior trochanter, separation from shaft: absent or minimal (0); present and extensive (1). (Nesbitt et al. 2009).

1444): Cervical vertebrae, lamina connecting posteroventral margin of diapophysis and posteroventral rim of centrum: absent (0); present (1). (Rauhut 2003).

1445): Premaxillary tooth 2, size relative to teeth 3 and 4: comparable (0); larger (1). (Senter 2010).

1447): Maxilla, lateral view, angle between anterodorsal and ventral margins: more than (0); less than (1) 80°. (Modified from Ezcurra and Novas 2007).

1450): Pedal phalanges, distal extensor pits, development: shallow (0); deep with defined margins (1). (Turner et al. 2009).

1452): Parietal, dorsal surface: flat (0); convex, posteroventrally directed (1).

1454): Astragalus, fibular facet, size: large, expanded anteroposteriorly (0); reduced, confined anteriorly (1). (Modified from Smith et al. 2008).

1455): Dentary, symphysis, medial surface, broad horizontal groove, anteriorly upturned and bounded ventrally by two ridges: absent (0); present (1).

1456): Tibia, lateral cnemial crest, dorsal/proximal margin, orientation: parallel to dorsal/proximal margin of medial condyle, or moderately upturned (0); strongly upturned (directed proximally) (1).

1457): Femur, proximal end, anterior trochanter major axis, angle formed with head major axis: more (0); less (1) than 45°. (Modified from Osi and Buffetaut 2011).

1458): Maxilla, subcutaneous flange bordering the antorbital fossa laterally on the posterior end of the main body, resulting in a fossa forming a channel between the flange and the main body: absent (0); present (1). (Brusatte et al. 2010).

1459): Jugal, lateral surface, cornual process/boss: absent (0); present (1).

1460): Postorbital, supraorbital (“cornual”) process, position: overhanging orbit (0); posteroventral to dorsal orbit margin (1).

1461): Femur, distal end, medial surface, longitudinal crest, distal bifurcation at the level of the medial condyle: absent (0); present (1). (Brusatte et al. 2010).

1462): Nasal, lateral surface, fossae/fenestrae, number: less than (0); more than (1) three.

1463): Sphenethmoid-orbitosphenoid contact: present (0); absent (1). (Paulina Carabajal and Currie 2012).

1464): Femur, posterior trochanter, position: along the posterolateral margin (0); centered on posterior surface (1). (Xu et al. 2012).

1465): Femur, shaft, medial margin, eminence just proximal to distal end: absent (0); present (1). (Xu et al. 2012).

1466): Mt II, shaft, distal end, anterior exposition: wide, broadly visible (0); barely visible due to plantar displacement (1) in articulated specimens. (Xu et al. 2012).

1467): Premaxillary and anterior dentary teeth, lingual surface, apicobasally oriented furrows/striations: absent (0); present (1).

1468): Skull, premaxilla-maxilla suture, lateral surface, subnarial foramen, shape: foramen (0); dorsoventrally directed channel (1). (Carrano et al. 2012).

1469): Maxilla, ventral process, lateral surface, lateral foramina, number of rows: less than two (0); two (1). (Carrano et al. 2012).

1470): Maxilla, jugal articulation, lateral shelf: absent (0); present (1). (Carrano et al. 2012).

1471): Jugal, lacrimal articulation, flange overlapping lacrimal: absent (0); present (1). (Carrano et al. 2012).

1472): Postorbital-squamosal articulation, shape: planar (0); helically spiralling along its length (1). (Carrano et al. 2012).

1473): Laterosphenoid-frontal articulation: present (0); absent (1). (Modified from Carrano et al. 2012).

1474): Prefrontal-frontal articular surface: planar (0); peg-in-socket (1). (Carrano et al. 2012).

1475): Quadrate, proximal end, shape in proximal view: rounded/oval (0); quadrangular (1). (Modified from Carrano et al. 2012).

1476): Paroccipital process, ventral margin, shape of curve described with stapedial groove/fenestra ovalis: broad arch (0); narrow/acute curve (1). (Carrano et al. 2012).

1477): Cervical vertebrae, anterior and middle centra, parapophysis, position: close to the anterior (0); close to middle (1) of centrum lateral surface. (Carrano et al. 2012).

1478): Dorsal vertebrae, anterior centra, pleurocoel, size: larger than a nutritive foramen but covering less than half of the lateral surface (0); hypertrophied, as large or larger than half lateral surface (1). (Carrano et al. 2012).

1479): Sacral vertebrae, neural spines, dorsal surface, mediolateral width: comparable (0); wider (1) than remainder of spine. (Carrano et al. 2012).

1481): Pubis, ischial peduncle, obturator perforation, longest axis, length: less (0); more than (1) ¾ of acetabular margin of pubis. (Modified from Carrano et al. 2012).

1482): Fibula, insertion of M. ileofibularis, shape: tubercle (0); anterolaterally curved flange (1). (Modified from Carrano et al. 2012).

1483): Humerus, deltopectoral crest, anterior margin, orientation: anterior (0); curved anterolaterally (1).

1484): Basisphenoid, indentation between basal tubera and basipterygoid processes, shape in lateral view: deep notch (0); shallow embayment (1). (Carrano and Sampson 2008).

1485): Premaxilla, ventral margin, shape in lateral view: straight to convex (0); concave (1).

1486): Scapula, anterior end, lateral surface, supraglenoid fossa, development: shallow, with poorly defined margins (0); deep, dorsoventrally oriented and with defined margins (1). (Pol and Rauhut 2012).

1487): Squamosal, participation to the nuchal crest: absent or minimal (0); extensive (1). (Pol and Rauhut 2012).

1488): Pubis-iliac articulation, facets, shape: planar (0); peg-in-socket (1).

1489): Mcs II and III, distal end, lateral condyle, ventrolateral process projected proximally: poorly developed (0); well-developed (1). (Modified from Pol and Rauhut 2012).

1490): Mc III, distal end, collateral shelves protruding dorsal to the collateral fossae: absent (0); present (1). (Ezcurra et al. 2010).

1491): Mc II and III, distal end, flexor fossa, development: deep with distinct margins (0); shallow, poorly defined (1). (Modified from Ezcurra et al. 2010).

1492): Humerus, proximal end, lateral tuberosity, development: well-developed, giving the lateral margin a straight profile in anterior/posterior view (0); reduced, giving the lateral margin a convex profile in anterior/posterior view (1). (Modified from Ezcurra et al. 2010).

1493): Frontal, prefrontal facet, position: extended posterolaterally, approaching the postorbital facet (0); limited anteromedially and excluded from orbital rim (1).

1494): Fronto-lacrimal suture, orientation in dorsal view: mainly anteroposteriorly (0); mainly mediolaterally (1).

1495): Frontal, supratemporal fossa, anteriormost margin, position: in the center or lateral half (0); in the medial half (1) of fossa. (Sampson and Carrano 2007).

1496): Frontal, prefrontal/lacrimal facet, dorsoventral thickening: absent (0); present (1).

1499): Manual ungual I, ventrodistal curvature in lateral view: marked (0); reduced (1).

1500): Frontal, nasal process, overlapping by nasal, extent: absent or limited (0); extensive, in particular medially, on almost or all the process dorsal surface (1).

1506): Mcs II-III, distal end, dorsoventrally oriented intercondylar sulcus, depth: deep, well-defined (0); shallow (1).

1507): Maxillary/post-symphyseal dentary tooth crowns, labial surface, shape: smooth (0); bearing apicobasally directed ridges/flutes (1). (Modified from Senter 2011).

1508): Maxillary teeth ventral to antorbital fenestra: present (0); absent (1).

1509): Maxillary teeth anterior to antorbital fenestra: present (0); absent (1).

1514): Jugal, postorbital (ascending) process, base, anteroposterior extent: narrow (length less than half process height) (0); broad (length more than half process height) (1).

1518): Jugal, anterior end, projection ventral to the antorbital fenestra, fitting into a maxillary cleft: absent (0); present (1).

1520): Dentary, lingual bar: present (0); absent (1).

1526): Dorsal vertebrae, postzygapophyses, orientation in posterior view: subhorizontal (0); medioventrally inclined (1). (Longrich and Currie 2009b).

1527): Postorbital, suborbital process, anterior margin, dorsoventral depth: shallow, process acuminate anteriorly (0); deep, process blunt anteriorly (1).

1528): Teeth, root, diameter along apicobasal axis: uniform (0); narrowing basally (1).

1530): Exoccipital, posterior surface, fossa housing the X and XII cranial nerves: absent (0); present (1). (Longrich and Currie 2009a).

1535): Mt IV, proximal end, lateral side, shape: flat (0); convex (1). (He et al. 2013).

1536): Frontal, prefrontal facet, shape in dorsal view: flat to arched (0); deep notch (1). (Loewen et al. 2013).

1537): Femur, distal end, lateral condyle, anterior bulge that is slightly separated from the remainder of the condyle: absent (0); present (1). (Brusatte et al. 2010).

1540): Basioccipital, basal tubera, dorsoventral depth: subequal or less (0); more (1) than occipital condyle midline depth. (Modified from Brusatte et al. 2010).

1541): Basioccipital, basal tubera, ventral notch between tubera, proportion in posterior view: wider than deep (0); as deep or deeper than wide (1).

1544): Cervical vertebrae, middle centra, centrodiapophyseal lamina, joining with centrum, position: in posterior half (0); in the middle or anterior half (1). (Farke and Sertich 2013).

1545): Dorsal vertebrae, middle and posterior centra, horizontal lamina bisecting the infradiapophyseal fossa: absent (0); present (1). (Farke and Sertich 2013).

1548): Ilium, preacetabular process, lateroventral fossa, rimmed margin: absent (0); present (1).

1549): Ilium, preacetabular process, ventral surface, posterior end: narrow (0); mediolaterally expanded (1).

1550): Posterior cervical and cervicodorsal vertebrae, transverse processes, large ventral foramina: absent (0); present (1).

1551): Maxilla, medial surface, medial antorbital fossa: absent (0); present (1).

1552): Tibia, cnemial crest, anterior end, lateral surface, proximodistally oriented ridge: absent (0); present (1).

1553): Humerus, deltopectoral crest, lateral surface, biceps scar along distal margin: absent (0); present (1).

1555): Premaxilla, narial fossa, development: slightly expanded anteroventrally to the external naris (0); extensive, covering almost the whole lateral surface of the premaxillary body (1). (Modified from Langer and Benton 2006).

1556): Maxilla, ascending process, pneumatic recesses, development: shallow fossa closed medially (0); medially open fenestra (1).

1557): Nasal, median crest/eminence, development: restricted to nasal mid-length (0); extending over most of the nasal (1).

1560): Frontal, dorsal surface, supratemporal fossa, extent: limited to the posterior third of the bone (0); extended for more than the posterior third of the bone (1). (Modified from Sereno 1999; Norell et al. 2001).

1561): Parietal, posterodorsal projection (nuchal plate), development: slightly developed (0); hypertrophied (1). (Wilson et al. 2003).

1563): Postorbital, suborbital process bordering ventrally the eyeball, development: anteroposteriorly reduced, long no more than 1/3 of the maximum orbital anteroposterior length (0); pronounced, long more than 1/3 of the maximum orbital anteroposterior length (1). (Modified from Wilson et al. 2003; Currie and Carpenter 2000).

1565): Quadratojugal, posteroventral process, length: no more than (0); more than (1) 1/2 of the anteroventral process of the quadratojugal. (Modified from Holtz et al. 2004).

1566): Basisphenoid, ventral recess, depth: shallow (0); deep (1). (Holtz 2001; Rauhut 2003).

1567): Dentary, posterodorsal process, length: less than (0); more than (1) 1/2 of the posteroventral process. (Modified from Holtz 2000).

1570): Surangular, laterodorsal shelf, depth: shallow (0); deep (1). (Modified from Holtz 2000).

1571): Prearticular, medial process, elongation: short (0); elongate (1). (Modified from Norell et al. 2001; Carr 2005).

1572): Axis, epipophyses, posterolateral extent: does not (0); does (1) overhang the postzygapophyses. (Rauhut 2003).

1573): Postaxial cervial prezygoepipophyseal lamina, development: low ridge (0); prominent (1). Ordered. (Modified from Carrano et al. 2002).

1574): Postaxial cervial epipophyses, development: small ridges (0); overhanging the postzygapophyses (1). (Rauhut 2003).

1576): Caudal vertebrae, anterior neural arches, ventral rib laminae, development: slightly developed (1); prominent and bordering deep fossae (1).

1578): Coracoid, tubercle, lateral development: slightly developed (0); strongly developed (1). (Modified from Holtz 2000).

1581): Ilium, antitrochanter, development: low and small (0); prominent (1).

1582): Ilium, postacetabular process, medioventral shelf, posterior half, lateral esposition: absent (0); present (1). (Modified from Carrano et al. 2002).

1583): Pubis, distal foot, anterior process, anteroposterior length: less (0); more (1) than 1/5 of the proximodistal length of the pubis. (Modified from Holtz 2000).

1584): Pubis, distal foot, posterior process, anteroposterior length: less than (0); more than (1) 1/5 of the length of the pubis. (Modified from Holtz 2000).

1588): Premaxilla, lateral surface pierced by neurovascular foramina, number: one, above the second premaxillary tooth (or in a comparable position in toothless forms) (0); several neurovascular foramina (1). (Modified from Ezcurra and Novas 2006).

1589): Nasal, pneumatisation development: unfenestrated (0); fenestrated (1).

1590): Postorbital, ventral process, ventral end, dorsoventrally oriented sulcus: absent, process single (0); present, process forked (1). (Novas et al. 2013).

1591): Cervical vertebrae, anterior and middle postzygodiapophyseal laminae, orientation in lateral view: posterodorsally (0); subvertical (1). (Modified from Novas et al. 2013).

1592): Dorsal ribs, pneumatic recess: absent (0); present (1). (Novas et al. 2013).

1593): Ulna, olecranon process, shape in lateral/medial view: quadrangular with subparallel dorsal and ventral margins (0); trapezoidal, distally tapering (1).

1595): Femur, distal view, angle between posterior margin of lateral condyle and lateral surface of ectocondylar tuber: wide, subequal or more than 90° (0); narrow, acute (1). (Novas et al. 2013).

1596): Mt III, distal end, extensor fossa, development: shallow and poorly defined (0); deep and crescentic in anterior view (1). (Novas et al. 2013).

1597): Femur, proximal end, lateral border, shape: broad rounded/squared (0); tapering (1). (Modified from Novas et al. 2013).

1598): Manual phalanx P1-I, shape in proximal view: triangular, broadest ventrally (0); quadrangular, dorsal surface as wide or wider than ventral (1). (Modified from Novas et al. 2013).

1599): Mt III, proximal epiphysis, mediolateral width: more (0); subequal or less (1) than shaft width. (Novas et al. 2013).

1600): Humerus, deltopectoral crest, proximoanterior margin, shape in medial/lateral view: straight to convex, crest confluent with level of head (0); concave, crest abruptly projected distal to head (1).

1602): Astragalus, distal end, anterior margin, lateral half, shape: slightly concave (0); markedly convex, anteriorly protruding (1). (Novas et al. 2013).

1603): Dentary, medial surface, Meckelian sulcus, dorsoventral depth: wide sulcus (0); narrow groove (1). (Brusatte et al. 2010).

1604): Cervical vertebrae, infrapostzygapophyseal fossa, position relative to posterior centrodiapophyseal lamina: fossa placed posterior to lamina (0); fossa placed dorsally to lamina (1). (Brusatte et al. 2010).

1605): Caudal vertebrae, anterior neural arches, anterolateral surface, deep triangular prezygocostal fossa delimited by two laminae: absent (0); present (1). (Modified from Brusatte et al. 2010).

1606): Mt III, distal end, lateral surface, large and rugose oval scar for mt IV: absent (0); present (1). (Brusatte et al. 2010).

1607): Femur, distal end, ectocondylar tuber, shape in distal view: rounded to quadrangular (0); kidney-shaped, concave posteriorly (1). (Modified from Novas et al. 2013).

1609): Jugal, posterior (infratemporal) process, depth: as deep or shallower (0); deeper (1) than suborbital process. (Carrano et al. 2012).

1610): Quadrate, dorsoventral axis of bone relative to mediolateral axis of distal condyles in posterior view: perpendicular (0); medially inclined (1). (Carrano et al. 2012).

1611): Posterior dorsal vertebrae, hyposphene, step-like ridges on lateral margin, development: short, poorly developed (0); long and prominent, bisecting the infrapostzygapophyseal fossa (1). (Modified from Carrano et al. 2012).

1612): Atlas, epipophyses, development: small (0); prominent (1). (Carrano et al. 2012).

1613): Cervical ribs, anterior process, length: short (0); prominent (1). (Carrano et al. 2012).

1614): Interdental septa in premaxilla and anterior dentary, spacing: regular (0); alternate (alveoli result paired) (1).

1620): Jugal, surface medial to the anterior pneumatic recess, shape: flat (0); excavated by a fossa (1). (Brusatte et al. 2010).

1621): Orbit, main plane orientation relative to anteroposterior axis of skull in articulated specimens: lateral (0); anterior, due to mediolateral expansion of postorbital region (1). (Modified from Loewen et al. 2013).

1622): Maxilla, palatal shelf, ventral surface, occlusal pits: absent (0); present (1). (Modified from Loewen et al. 2013).

1623): Squamosal, postquadratic process, lateral view, anteroposterior width perpendicular to main proximodistal axis: less (0); more (1) than bone length. (Modified from Loewen et al. 2013).

1624): Jugal, suborbital process at base of postorbital process: absent (0); present (1). (Modified from Loewen et al. 2013).

1625): Quadratojugal, anterior process, dorsoventral expansion overlapping the jugal laterally: absent (0); present (1). (Loewen et al. 2013).

1626): Supraoccipital, dorsal process, dorsal surface, shape: unforked (0); forked (1). (Modified from Loewen et al. 2013).

1627): Palatine, anterior/vomerine process: absent/indistinct (0); present/distinct (1). (Loewen et al. 2013).

1628): Prearticular, shape in lateral/medial view: gently concave dorsally (0); “U”-shaped (1). (Loewen et al. 2013).

1630): Prearticular, mid-shaft cross section, proportions: dorsoventrally taller than mediolaterally wide (0); as wide as tall (1). (Modified from Loewen et al. 2013).

1631): Fibula, shaft distal to M. ileofibularis insertion, medial surface, shape: flat to convex (0); concave due to presence of groove (1). (Loewen et al. 2013).

1632): Ilium, postacetabular process, brevis fossa, pneumatic foramen: absent (0); present (1). (Zanno and Makovicky 2013).

1637): Scapula, ventral margin just distal to glenoid, shape in lateral/medial view: straight to convex (0); with a distinct concavity (1). (Tortosa et al. 2013).

1638): Tibia, fibular crest, orientation: straight, proximodistal (0); curved anteroproximally (1). (Modified from Tortosa et al. 2013).

1639): Prootic, tuberosity on the margin of the crista prootica (otosphenoid crest): absent (0); present (1). (Tortosa et al. 2013).

1640): Quadrate, anterior surface, large funnel-like recess: absent (0); present (1). (Brusatte et al. 2010).

1642): Maxilla, ventral process, posterior extent relative to the lacrimal (preorbital) bar: posterior (0); ventral (1).

1644): Coracoid, infraglenoid buttress placed ventrodistal to glenoid, eventually forming a cleft with posteroventral process: absent (0); present (1).

1655): Dentary, lateral surface, anterior half, accessory longitudinal groove placed just above ventral margin: absent (0); present (1).

1657): Sacrum: primordial sacral 1 and 2, relationships: adjacent (0); separated by one or more “insertion” vertebrae (1).

1659): Maxilla, maxillary fenestra, area in lateral view: less (0); more (1) than half of surface of anterior antorbital fossa.

1661): Maxilla, ascending ramus, lateral surface, dorsoventrally oriented neurovascular groove (“anterior groove” of Sereno et al. 2004): absent (0); present (1).

1663): Maxilla, lateral surface, jugal ramus, anastomosing pattern of grooves (“posterior groove” of Sereno et al. 2004): absent (0); present (1).

1670): Teeth, enamel microstructure, boundary between first and second enamel types from the enamel-dentine junction: parallel to enamel-dentine junction (0); jagged, varies in distance from enamel-dentine (1). (Hwang 2007; Hendrickx and Mateus 2014a).

1675): Maxilla, ascending process, posterodorsal end, shape in lateral view: single (0); forked (1).

1676): Maxilla/dentary, paradental laminae, shape in medial view: triangular, apically acuminate (0); quadrangular, apically flat (1). (Hendrickx and Mateus 2014b).

1682): Maxillary/dentary teeth, mesial carina, denticle at 2/3 of the carina, proportions: longer apicobasally than mesiodistally (0); mesiodistally as wide or wider than apicobasally long (1). (Modified from Hendrickx and Mateus 2014a).

1683): Maxillary/dentary teeth, distal carina, denticle at 1/2 of the carina, proportions: as long or longer apicobasally than mesiodistally (0); mesiodistally wider than apicobasally long (1). (Modified from Hendrickx and Mateus 2014a).

1684): Ulna, proximal view: orientation of olecranon process of the ulna: in same plane as coronoid (anterior/sigmoid) process (0); significantly everted medially, angle between olecranon and coronoid processes close to 120°(1). (Smith et al. 2008).

1693): Distal carpal 1+2, proximal articular surface, transverse groove: absent (0); present at least medially (1). (Modified from Xu et al. 2014).

1694): Distal carpal 3: present (0); absent (1). (Modified from Xu et al. 2014).

1695): Distal carpal 4: present (0); absent (1). (Modified from Xu et al. 2014).

1696): Distal carpal 1+2, dorsomedial process, development: indistint (0); prominent (1). (Modified from Xu et al. 2014).

1702): Maxilla, facet for subnarial process of premaxilla, position: lateral to anteromedial process, on lateral surface (0); into a slot between anteromedial process and lateral surface (1). (Modified from Wilson et al. 2003).

1703): Premaxilla-maxilla suture, upper portion immediately ventral to the maxillary process of premaxilla, pneumatic spaces: absent (0); present (1). (Modified from Wilson et al. 2003).

1704): Maxilla, palatal shelves, anteroposterior extent: elongate for most of buccal margin (0); short (1).

1705): Lacrimal, anterodorsal process: present (0); absent (1).

1706): Exocipital, fossa for cranial nerves X-XII, depth: shallow, bowl-like (0); deep funnel (1). (Modified from Brusatte et al. 2010).

1707): Fourth sacral vertebra: absent (0); present (1).

1708): Fifth sacral vertebra: absent (0); present (1).

1709): Sixth sacral vertebra: absent (0); present (1).

1715): Manual digit 3, non-ungual phalanges, number: three (0); less than three (1).

1717): Premaxilla, sixth alveolus: absent (0); present (1).

1718): Premaxilla, seventh alveolus: absent (0); present (1).

1723): Mc III, distal end, position relative to mc II: distal (0); not distal (1).

1732): Mc II and III, contact along shafts: limited proximally (0); extended for all shaft (1).

1736): Maxilla, palatal shelf, dorsoventral thickness: shallow shelf (0); thick torus maxillaris confluent with anteromedial process (1).

1740): Dorsal vertebrae, anterior parapophyses, size: less (0); more (1) than half depth of anterior facet of centrum. (Brusatte et al., 2014).

1741): Scapula, anterior end, lateral surface dorsal to glenoid, distinct ridge overhanging fossa: absent (0); present (1). (Modified from Brusatte et al., 2014).

1742): Femur, head, anterior surface, anteroposteriorly (horizontally) oriented ridge overhaning distinct fossa/sulcus: absent (0); present (1).

1743): Femur, fourth trochanter, proximal end, confluence with greater trochanter: absent (0); present (1). (Modified from Brusatte et al., 2014).

1746): Quadrate, quadrate ridge, inclination in posterior view: laterally inclined (0); subvertical (1). (Hendrickx et al., 2015).

1747): Quadrate, quadrate ridge, contact with lateral condyle: asbent (0); present (1). (Modified from Hendrickx et al., 2015).

1748): Quadrate, quadrate ridge, ventral extremity, bifurcation: absent (0); present (1). (Hendrickx et al., 2015).

1749): Quadrate, quadrate ridge, lateral protuberance at 2/3 of ridge: absent (0); present (1). (Hendrickx et al., 2015).

1750): Quadrate, distal end, anterior intercondylar notch: absent (0); present (1). (Modified from Hendrickx et al., 2015).

1751): Quadrate, medial condyle, anterior concavity: absent (0); present (1). (Modified from Hendrickx et al., 2015).

1752): Quadrate, intercondylar sulcus, width: less (0); subequal or larger (1) than medial condyle. (Hendrickx et al., 2015).

1753): Quadrate foramen, shape: not elongate dorsoventrally (0); elongate dorsoventrally (1). (Modified from Hendrickx et al., 2015).

1754): Quadrate, lateral process: present (0); absent (1). (Hendrickx et al., 2015).

1755): Quadrate, lateral process, dorsal extent: reaching (0); not reaching (1) the quadrate head. (Hendrickx et al., 2015).

1759): Premaxilla, fourth alveolus: absent (0); present (1).

References

Agnolin, F.A., Powell, J.E., Novas, F.E, and Kundrát, M. (2012). New alvarezsaurid (Dinosauria, Theropoda) from uppermost Cretaceous of north–western Patagonia with associated eggs. Cretaceous Research 35: 33–56.

Barrett, P.M. (2009). The affinities of the enigmatic dinosaur *Eshanosaurus deguchiianus* from the Early Jurassic of Yunnan Province, People's Republic of China. Palaeontology, 52(4): 681–688.

Benson R.B.J., Brusatte S.L. and Carrano, M.T. (2010). A new clade of large–bodied predatory dinosaurs (Theropoda: Allosauroidea) that survived to the latest Mesozoic. Naturwissenschaften. 97: 71–78.

Brusatte, S.L., Benson, R.B.J. and Xu, X. (2012). A reassessment of *Kelmayisaurus petrolicus*, a large theropod dinosaur from the Early Cretaceous of China. Acta Palaeontologica Polonica 57(1): 65–72.

Brusatte, S.L. and Sereno, P.C. (2008). Phylogeny of Allosauroidea (Dinosauria: Theropoda): comparative analysis and resolution. Journal of Systematic Palaeontology, 6: 155-182.

Brusatte, S. L., Norell, M.A., Carr, T.D., Erickson, G.M., Hutchinson, J.R., Balanoff, A.M., Bever, G.S., Choiniere, J.N., Makovicky, P.J. and Xu, X. (2010). Tyrannosaur paleobiology: new research on ancient exemplar organisms. Science, 329: 1481−1485.

Brusatte, S.L., Lloyd, G.T., Wang, S.C., Norell, M.A. 2014. Gradual assembly of avian body plan culminated in rapid rates of evolution across the dinosaur-bird transition, Current Biology http://dx.doi.org/10.1016/j.cub.2014.08.034

Carrano, M.T. and Sampson, S.D. (2008). The phylogeny of Ceratosauria (Dinosauria: Theropoda). Journal of Systematic Paleontology, 6: 183–236.

Carrano, M.T., Benson, R.B.J. and Sampson, S.D. (2012). The phylogeny of Tetanurae (Dinosauria: Theropoda). Journal of Systematic Palaeontology 10(2): 211-300.

Cau, A., Brougham, T. and Naish, D. (2015). The phylogenetic affinities of the bizarre Late Cretaceous Romanian theropod *Balaur bondoc* (Dinosauria, Maniraptora): dromaeosaurid or flightless bird? PeerJ 3:e1032; DOI 10.7717/peerj.1032

Choiniere, J.N., Clark, J.M., Forster, C.A, and Xu, X. (2010). A basal coelurosaur (Dinosauria: Theropoda) from the Late Jurassic (Oxfordian) of the Shishugou Formation in Wucaiwan, People's Republic of China. Journal of Vertebrate Paleontology 30(6): 1773–1796.

Coria, R.A. and Currie, P.J. (2002). Braincase of *Giganotosaurus carolinii* (Dinosauria: Theropoda) from the Upper Cretaceous of Argentina. Journal of Vertebrate Paleontology, 22(4): 802–811.

Coria, R.A. and Salgado, L. (2000). A basal Abelisauria Novas, 1992 (Theropoda–Ceratosauria) from the Cretaceous of Patagonia, Argentina. Gaia 15: 89–102.

Currie, P.J. and Carpenter, K. (2000). A new specimen of *Acrocanthosaurus atokensis* (Theropoda, Dinosauria) from the Lower Cretaceous Antlers Formation (Lower Cretaceous, Aptian) of Oklahoma, USA. Geodiversitas 22 (2): 207–246.

Eddy, D.R. and Clarke, J.A. (2011). New information on the cranial anatomy of *Acrocanthosaurus atokensis* and its implications for the phylogeny of Allosauroidea (Dinosauria: Theropoda). PLoS One 6 (3): e17932.

Ezcurra, M.D. and Brusatte, S.L. (2011). Taxonomic and phylogenetic reassessment of the early neotheropod dinosaur *Camposaurus arizonensis* from the Late Triassic of North America. Palaeontology 54 (4): 763–772.

Ezcurra, M.D. and Novas, F.E. (2007). Phylogenetic relationships of the Triassic theropod *Zupaysaurus rougieri* from NW Argentina. Historical Biology 19(1): 35-72.

Ezcurra, M.D., Agnolin, F.L. and Novas, F.E. (2010). An abelisauroid dinosaur with a non–atrophied manus from the Late Cretaceous Pari Aike Formation of southern Patagonia. Zootaxa 2450: 1–25.

Farke, A.A. and Sertich, J.J.W. (2013). An abelisauroid theropod dinosaur from the Turonian of Madagascar. PLoS One 8(4): e62047.

Gauthier, J. (1986). Saurischian monophyly and the origin of birds. Memoirs of the California Academy of Sciences, 8: 1–55.

Gishlick, A.D. (2002). The functional morphology of the forelimb of *Deinonychus antirrhopus* and its importance for the origin of avian flight. PhD Dissertation. Yale University.

Grellet–Tinner, G. and Makovicky, P. (2006). A possible egg of the dromaeosaur *Deinonychus antirrhopus*: phylogenetic and biological implications. Canadian Journal of Earth Sciences, 43: 705–719.

He, Y.-M., Clark, J.M. and Xu, X. (2013). A large theropod metatarsus from the upper part of the Jurassic Shishugou Formation in Junggar Basin, Xinjiang, China. Vertebrata PalAsiatica 51: 29–42.

Hendrickx, C. and Mateus, O. (2014a). Abelisauridae (Dinosauria: Theropoda) from the Late Jurassic of Portugal and dentition-based phylogeny as a contribution for the identification of isolated theropod teeth. Zootaxa 3759: 1–74.

Hendrickx, C. and Mateus, O. (2014b). *Torvosaurus gurneyi* n. sp., the largest terrestrial predator from Europe, and a proposed terminology of the maxilla anatomy in nonavian theropods. PLoS One 9(3): e88905.

Hendrickx, C., Mateus, O. and Buffetaut, E. (2016). Morphofunctional analysis of the quadrate of Spinosauridae (Dinosauria: Theropoda) and the presence of *Spinosaurus* and a second spinosaurine taxon in the Cenomanian of North Africa. PLoS ONE 11(1): e0144695. doi:10.1371/journal.pone.0144695

Holtz, T.R., Jr. (2000). A new phylogeny of the carnivorous dinosaurs. Gaia 15, 5–61.

Holtz, T.R., Jr., Molnar, R.E. and Currie, P.J. (2004). The Dinosauria (second edition) (eds. Weishampel, D. B., Dodson, P. and Osmolska, H. ) pp. 71–110 (University of California Press, Berkeley).

Hwang, S.H. (2007). Phylogenetic patterns of enamel microstructure in dinosaur teeth. Unpublished Ph.D. dissertation, Columbia University, New York, 274 pp.

Lamanna, M.C., Sues, H.-D., Schachner, E.R. and Lyson, T.R. (2014). A new large-bodied oviraptorosaurian theropod dinosaur from the latest Cretaceous of western North America. PLoS One 9(3): e92022.

Langer, M.C. and Benton, M.J. (2006). Early dinosaurs: a phylogenetic study. Journal of Systematic Palaeontology 4(4): 309-335.

Lee, M.S.Y., Cau, A., Naish, D. and Dyke, G.J. (2014). Sustained miniaturization and anatomical innovation in the dinosaurian ancestors of birds. Science 345(6196): 562–566.

Loewen, M.A., Irmis, R.B., Sertich, J.J.W., Currie, P.J. and Sampson, S.D. (2013). Tyrant dinosaur evolution tracks the rise and fall of Late Cretaceous oceans. PLoS One 8 (11): e79420.

Longrich, N.R. and Currie, P.J. (2009a). A microraptorine (Dinosauria–Dromaeosauridae) from the Late Cretaceous of North America. Proceedings of the National Academy of Sciences 106(13): 5002–5007.

Longrich, N.R. and Currie, P.J. (2009b). *Albertonykus borealis*, a new alvarezsaur (Dinosauria: Theropoda) from the Early Maastrichtian of Alberta, Canada: Implications for the systematics and ecology of the Alvarezsauridae. Cretaceous Research 30 (1): 239–252.

Longrich, N.R., Currie, P.J. and Dong, Z.–M. (2010). A new oviraptorid (Dinosauria: Theropoda) from the Upper Cretaceous of Bayan Mandahu, Inner Mongolia. Palaeontology 53(5): 945–960.

Lü, J., Currie, P.J., Xu, L., Zhang, X., Pu, H. and Jia, S. (2013) Chicken-sized oviraptorid dinosaurs from central China and their ontogenetic implications. Naturwissenschaften (advance online publication) DOI: 10.1007/s00114-012-1007-0

Madsen, J.H. (1976). Allosaurus fragilis: A Revised Osteology. Utah Geological Survey Bulletin 109 (2nd ed.). Salt Lake City: Utah Geological Survey.

Makovicky, P.J., Apesteguía, S. and Agnolín, F.L. (2005). The earliest dromaeosaurid theropod from South America. Nature, 437; 1007–1011.

Martínez, R.N., Sereno, P.C., Alcober, O.A., Colombi, C.E., Renne, P.R., Montañez, I.P. and Currie, B.S. (2011). A basal dinosaur from the dawn of the dinosaur era in southwestern Pangaea. Science 331(6014): 206–210.

Maryańska, T., Osmólska, H. and Wolsan, M. (2002). Avialan status for Oviraptorosauria. Acta Palaeontologica Polonica 47(1): 97–116.

Nesbitt, S.J., Turner, A.H., Spaulding, M., Conrad, J.L. and Norell, M.A. (2009). The theropod furcula. Journal of Morphology, 270: 856-879.

Nesbitt, S.J., Sidor, C.A., Irmis, R.B., Angielczyk, K.D., Smith, R.M.H. and Tsuji, L.M.A. (2010). Ecologically distinct dinosaurian sister group shows early diversification of Ornithodira. Nature 464 (7285): 95–98.

Novas, F.E., Ezcurra, M.D. and Lecuona, A. (2008). *Orkoraptor burkei* nov. gen. et sp., a large theropod from the Maastrichtian Pari Aike Formation, Southern Patagonia, Argentina. Cretaceous Research 29 (3): 468–480.

Novas, F.E., Agnolín, F.L., Ezcurra, M.D., Porfiri, J. and Canale, J.I. (2013). Evolution of the carnivorous dinosaurs during the Cretaceous: The evidence from Patagonia. Cretaceous Research 45: 174–215.

O’Connor, J.K. (2009). A Systematic Review of Enantiornithes (Aves: Ornithothoraces). Unpublished Ph.D. thesis, University of Southern California, Los Angeles, 406 pp.

Osi, A. and Buffetaut, E. (2011). Additional non-avian theropod and bird remains from the early Late Cretaceous (Santonian) of Hungary and a review of the European abelisauroid record, Annales De Paleontologie, 97(1-2): 35-49.

Paulina Carabajal, A. and Currie, P.J. (2012). New information on the braincase of *Sinraptor dongi* (Theropoda: Allosauroidea): ethmoidal origin, endocranial anatomy and pneumaticity, Vertebrata PalAsiatica, 50(2): 85-101.

Pol, D. and Rauhut, O.W.M. (2012). A Middle Jurassic abelisaurid from Patagonia and the early diversification of theropod dinosaurs. Proceedings of the Royal Society B: Biological Sciences 279(1804): 3170–5.

Rauhut, O.W.M. (2003). The interrelationships and evoluton of basal theropod dinosaurs. Special papers in Palaeontology 69 (Palaeontological Association, London).

Rauhut, O.W.M. (2011). Theropod dinosaurs from the Late Jurassic of Tendaguru (Tanzania). Special Papers in Palaeontology 86: 195-239.

Rauhut, O.W.M. and Xu, Xing (2005). The small theropod dinosaurs *Tugulusaurus* and *Phaedrolosaurus* from the Early Cretaceous of Xinjiang, China. Journal of Vertebrate Paleontology 25(1): 107–118.

Senter, P. (2007). A new look at the phylogeny of Coelurosauria (Dinosauria: Theropoda). Journal of Systematic Palaeontology, 5: 429–463.

Senter, P. (2011). Using creation science to demonstrate evolution 2: morphological continuity within Dinosauria. Journal of Evolutionary Biology, 24(10): 2197–2216.

Sereno, P.C. (1999). The evolution of dinosaurs. Science, 284: 2137–2147.

Sereno, P.C., Wilson, J.A. and Conrad, J.L. (2004). New dinosaurs link southern landmasses in the Mid-Cretaceous. Proceedings: Biological Sciences. 271(1546): 1325-1330.

Smith, N.D., Makovicky, P.J., Agnolín, F.L., Ezcurra, M.D., Pais, D.F. and S. W. Salisbury (2008). A Megaraptor–like theropod (Dinosauria:Tetanurae) in Australia: support for faunal exchange across eastern and western Gondwana in the Mid–Cretaceous. Proceedings of the Royal Society B, 275: 2085–2093.

Sues, H.–D., Nesbitt, S.J., Berman, D.S. and Henrici, A.C. (2011). A late–surviving basal theropod dinosaur from the latest Triassic of North America. Proceedings of the Royal Society B 278 (1723): 3459–3464.

Sullivan, C., Hone, D.W.E., Xu, X. and Zhang, F. (2010). The asymmetry of the carpal joint and the evolution of wind folding in maniraptoran theropod dinosaurs. Proceedings of the Royal Society B, 277: 2027–2033.

Tortosa, T., Buffetaut, E., Vialle, N., Dutour, Y., Turini, E. and Cheylan, G. (2013). A new abelisaurid dinosaur from the Late Cretaceous of southern France: Palaeobiogeographical implications. Annales de Paléontologie 100(1): 63-86.

Turner, A.H., Hwang, S.H. and Norell, M.A. (2007). A small derived theropod from Öösh, Early Cretaceous, Baykhangor Mongolia. American Museum Novitates 3557: 1–27.

Turner, A.H., Makovicky, P.J. and Norell, M.A. (2012). A review of dromaeosaurid systematics and paravian phylogeny. Bulletin of the American Museum of Natural History 371: 1–206.

Turner, A.H., Nesbitt, S.J. and Norell, M.A. (2009a). A large alvarezsaurid from the Cretaceous of Mongolia. American Museum Novitates 3648: 1–14.

Tykoski, R.S. (2005). Anatomy, ontogeny, and phylogeny of coelophysoid theropods. PhD dissertation, University of Texas at Austin.

Wilson, J.A., Sereno, P.C., Srivastava, S., Bhatt, D.K., Khosla, A. and Sahni, A. (2003). A new abelisaurid (Dinosauria, Theropoda) from the Lameta Formation (Cretaceous, Maastrichtian) of India. Contributions from the Museum of Paleontology [University of Michigan] 31(1): 1–42.

Xu, X., Zhao, Q., Norell, M., Sullivan, C., Hone, D., Erickson, G., Wang, X., Han, F. and Guo, Y. (2009). A new feathered maniraptoran dinosaur fossil that fills a morphological gap in avian origin. Chinese Science Bulletin 54(3): 430–435.

Xu, X., Zhao, J., Sullivan, C., Tan, Q.-W., Sander, M. and Ma Q.-Y. (2012). The taxonomy of the troodontid IVPP V 10597 reconsidered. Vertebrata PalAsiatica 50(2): 140–150.

Yates, A.M. (2006). A new theropod dinosaur from the Early Jurassic of South Africa and its implications for the early evolution of theropods. Palaeontologia africana, 41: 105–122.

Zanno L.E, Varricchio D.J., O’Connor P.M., Titus A.L. and Knell M.J. (2011). A new troodontid theropod, *Talos sampsoni* gen. et sp. nov., from the Upper Cretaceous Western Interior Basin of North America. PLoS One, 6(9): e24487.

Zelenitsky, D.K., Therrien, F. and Kobayashi, Y. (2009). Olfactory acuity in theropods: palaeobiological and evolutionary implications. Proceeding of the Royal Society B, 276: 667–673.
